# Sorlinia euscelidii gen. nov., sp. nov., a novel acetic acid bacterium isolated from the leafhopper Euscelidius variegatus (Hemiptera: Cicadellidae)

**DOI:** 10.1099/ijsem.0.006544

**Published:** 2024-10-21

**Authors:** Ramona Marasco, Grégoire Michoud, Kholoud A. Seferji, Elena Gonella, Elisa Garuglieri, Eleonora Rolli, Alberto Alma, Francesca Mapelli, Sara Borin, Daniele Daffonchio, Elena Crotti

**Affiliations:** 1 Biological and Environmental Sciences and Engineering Division (BESE), King Abdullah University of Science and Technology (KAUST), Thuwal, Saudi Arabia; 2 Department of Agricultural, Forest, and Food Sciences (DISAFA), University of Torino, Turin, Italy; 3 Department of Food, Environmental and Nutritional Sciences (DeFENS), University of Milan, Milan, Italy

**Keywords:** acetic acid bacteria, *Acetobacteraceae*, *Euscelidius variegatus*, insect, *Sorlinia*

## Abstract

Acetic acid bacteria – belonging to the *Acetobacteraceae* family – are found in the gut of many sugar-feeding insects. In this study, six strains have been isolated from the hemipteran leafhopper *Euscelidius variegatus*. While they exhibit high 16S rRNA gene sequence similarities to uncultured members of the *Acetobacteraceae* family, they could not be unequivocally assigned to any particular type species. Considering the clonality of the six isolates, the EV16P^T^ strain was used as a representative of this group of isolates. The genome sequence of EV16P^T^ is composed of a 2.388 Mbp chromosome, with a DNA G+C content of 57 mol%. Phylogenetic analyses based on the 16S rRNA gene sequence and whole-genome multilocus sequence analysis indicate that EV16P^T^ forms a monophyletic clade with the uncultivated endosymbiont of *Diaphorina citri*, the *Candidatus* Kirkpatrickella diaphorinae. Such a phylogenetic clade is positioned between those of *Asaia-Swaminathania* and *Kozakia*. The genomic distance metrics based on gene and protein sequences support the proposal that EV16P^T^ is a new species belonging to a yet-undescribed genus. It is a rod-shaped Gram-stain-negative bacterium, strictly aerobic, non-motile, non-spore-forming, showing optimal growth without salt (NaCl) at 30 °C and pH of 6–7. The major quinone is Q10, and the dominant cellular fatty acids (>10%) are C_18:l_*ω*7c, C_19 : 0_ cyclo *ω*6c, C_16 : 0_ and C_19 : 1_ 2OH. The polar lipid profile comprises diphosphatidylglycerol, phosphatidylethanolamine and phosphatidylcholine, along with unidentified aminophospholipids, glycophospholipids, aminolipids and lipids. Based on a polyphasic approach, including phylogenetic, phylogenomic, genome relatedness, phenotypic and chemotaxonomic characterisations, EV16P^T^ (= KCTC 8296^T^, = DSM 117028^T^) is proposed as a representative of a novel species in a novel genus with the proposed name *Sorlinia euscelidii* gen. nov., sp. nov., in honour of Prof. Claudia Sorlini, an Italian environmental microbiologist at the University of Milan who inspired the research on microbial diversity, including symbiosis in plants and animals.

## Background

The interactions between insects and micro-organisms have shaped a complex mosaic of coevolution, adaptation and ecological interdependence, spanning various symbiotic relationships as either primary (obligate and essential for the insect’s development and long-term associations) or secondary (facultative, gained or lost over evolutionary time) [1,2]. These symbiotic partnerships profoundly influence host physiology and behaviour by impacting nutritional supplementation, defence against pathogens and niche adaptation [1]. Symbiosis also contributes to insect diversification and their remarkable evolutionary success across diverse habitats [1].

In insect–microbe interactions, acetic acid bacteria (AAB) have become a focal point of attention [3]. Historically known for their role in natural food fermentations, AAB within the *Acetobacteraceae* family [4] have emerged as intriguing symbionts of insects relying on sugar-based diets such as nectar, fruit sugars or phloem sap [1,3]. The widespread presence, prevalence and localization of AAB across both holometabolous (*Diptera*, *Hymenoptera*, *Blattodea* and *Coleoptera*) and hemimetabolous (*Hemiptera*) insect lineages have been established primarily based on molecular tools, including DNA sequencing of total bacterial communities (16S rRNA gene amplification and metagenome) and fluorescence *in situ* hybridization [5,9]. Recent efforts allowed the cultivation of several fastidious AAB [10], including (among others) *Acetobacter*, *Gluconobacter*, *Komagataeibacter*, *Gluconacetobacter, Asaia*, *Bombella* and *Entomobacter* strains from holometabolous insects within *Diptera*, *Hymenoptera* and *Blattodea* [5,11,25]. However, all the attempts to isolate AAB from *Hemiptera* have thus far proved unsuccessful, including the effort to cultivate a novel AAB endosymbiont identified by deep sequencing in *Diaphorina citri* [9]. The heightened fastidious nature of AAB in the association with hemipterans may be caused by a longstanding/ancient and strongly specialized symbiotic co-evolution with their host [5], which separated ~300 million years ago from the Holometabola [26]. In such instances, symbionts often exhibit reduced genomes, retaining only essential genes necessary for their survival [27], consequently limiting their capacity to adapt under different (environmental) conditions, like those occurring under laboratory cultivation, where the ‘host niche’ conditions can only be partially mimicked.

The cultivation and description of insect-associated AAB in hemipterans will help to shed light on their (co)evolution, physiology and ecological function during their association with this group of insects. To increase the chance of isolating such fastidious bacteria, we applied enrichment cultivation strategies starting from the two hemipterans, *Scaphoideus titanus* and *Euscelidius variegatus*. AAB isolation was performed from more than 50 individuals, considering the abovementioned species collected at different instars and using several AAB media [5,6] (Table S1, available in the online Supplementary Material). AAB are typically isolated through a procedure involving growth in an enrichment liquid medium, followed by plating on a calcium carbonate solid medium once turbidity is observed in the initial enrichment. These media commonly include sugars or polyols as a suitable carbon source and are adjusted to a pH of ~3.5. Notably, these media may contain acetic acid, which can be detrimental to most non-AAB species [28]. However, when applying these isolation strategies with *S. titanus*, we mainly recorded either the absence of bacterial growth in the enrichment step or the isolation of bacteria other than AAB. We found an exception with the leafhopper *E. variegatus*, from which six isolates were obtained and identified as AAB related to *Asaia* species; these isolates could not be assigned unequivocally to any validly published type strain, including those in the *Asaia* genus. Here, we describe the genomic, physiological and metabolic features of their representative strain EV16P^T^ as part of a newly proposed AAB genus thus far exclusively found in hemipterans (isolated from *E. variegatus* in this work and with a highly similar genome detected as *Candidatus* in *D. citri* [9]) and for which the name *Sorlinia euscelidii* gen. nov., sp. nov. is proposed in honour of the Italian microbiologist Prof. Claudia Sorlini.

## Isolation and ecology

*E. variegatus* Kirschbaum, an insect of the *Cicadellidae* family within the *Hemiptera* order, is a species of leafhoppers commonly found in wild and agroecosystems in Europe. It is a polyphagous species that feeds on phloem sap from host plants belonging to several families [29]. The specimens used in this work as starting material for AAB isolation had been collected from a long-term (20 years) laboratory mass rearing maintained at the Department of Agricultural, Forest, and Food Sciences (DISAFA) laboratories of the University of Turin, Italy. Insects were reared on oat plants (*Avena sativa* L.) in growth chambers at 25 °C with a photoperiod of 16 : 8 light:dark [30]. A total of ten adult specimens were collected and surface-sterilized by washing them with 1% sodium hypochlorite for 2 min, followed by a rinse with saline solution (9 g l^–1^ NaCl) for 5 min and a final wash in saline solution. Four insects were used as single specimens, while the other six were grouped in two pools of three individuals. All samples were homogenized in 200 µl of saline solution with a sterile micropestle. Twenty microlitres of the homogenates were inoculated in the enrichment ABEM medium [6] prepared with 2% (w/v) sorbitol, 0.5% (w/v) peptone, 0.3% (w/v) yeast extract, pH 3.5 and 100 µg ml^−1^ cycloheximide to inhibit fungi growth, following the procedure described for the isolation of *Asaia* first from flowers [31] and later from mosquitoes [14]. Other media, including TA1 [1% (w/v) glucose, 0.5% (v/v) ethanol, 0.3% (v/v) acetic acid, 1.5% (w/v) peptone, 0.8% (w/v) yeast extract, pH 3.5], YE [2% (w/v) yeast extract, 2% (v/v) ethanol, 1% (v/v) acetic acid, pH 6] and MAN agar plate [0.5% (w/v) mannitol, 0.3% (w/v) peptone, 0.5% (w/v) yeast extract, pH 7], have been used in the attempt to enrich and isolate AAB (Table S1). Inoculated media were incubated aerobically at 150 r.p.m. at 30 °C. When enrichment cultures showed bacterial growth (i.e. medium turbidity), they were subsequently spread onto modified GYCE (glucose–yeast extract–CaCO_3_–ethanol [32]) agar plates containing 2% (w/v) glucose, 0.8% (w/v) yeast extract, 0.7% (w/v) CaCO_3_, 0.5% (v/v) ethanol and 1.2% (w/v) agar, pH 6.8–7, or MA prepared with 1% (w/v) glucose, 1% (v/v) glycerol, 1% (w/v) peptone, 0.5% (w/v) yeast extract, 0.7% (w/v) CaCO_3_, 1% (v/v) ethanol and 1.5% agar, pH 6.8 (Table S1). Plates were then incubated aerobically at 30 °C, and bacterial growth was checked daily. Seven days later, the grown colonies were picked from agar media and individually streaked, and the procedure was repeated three times to purify them. The obtained bacterial isolates were stored in 20% sterile glycerol at −80 °C.

## 16S rRNA gene sequence phylogenetic analysis

The 16S rRNA gene of the obtained isolates (*n*=20) was amplified and sequenced at Macrogen Inc. (South Korea, www.macrogen.com) using the standard primers 27F (5′ AGA GTT TGA TCM TGG CTC AG 3′) and 1492R (5′ GGT TAC CTT GTT ACG ACT T 3′) [33,34]. Following nucleotide trimming based on quality (error <1%), forward and reverse reads were assembled into a single consensus sequence (~1325 bp fragments). After comparing the 16S rRNA gene sequences of our isolates with those available in the 16S rRNA gene sequence records of the National Center for Biotechnology Information (NCBI), we found that six isolates (namely EV15G, EV15P, EV16GL, EV16GM, EV16P and EV17) were closely related to an uncultivated *Asaia* sp. (MN099438, 100% of nucleotide identity) and the recently described uncultivated endosymbiont *Ca*. Kirkpatrickella diaphorinae (OP600170.1 [9]; 99.7% of nucleotide identity) but could not be identified without ambiguity to any validly described species within the ‘type material’ rRNA/ITS database (Fig. S1). The six isolates were all obtained from one ABEM enrichment, inoculated with the homogenate obtained from a single individual, with final plating on GYCE (Table S1). The most closely related 16S rRNA gene sequences of type strains from LPSN (List of Prokaryotic names with Standing in Nomenclature [4]) belong to *Asaia lannensis* (NR_114144; 97.2% identity), *A. bogorensis* and *A. prunellae* (NR_113849 and NR_112880, 97.1%), *A. krungthepensis* (NR_113878, 97%), *A. platycodi* (NR_112879, 97%), *A. spathodeae* (NR_114292, 97%), *Swaminathania salitolerans* (NR_025217, 96.4%) and *Kozakia baliensis* (NR_113858, 96.2%). The low 16S rRNA gene sequence identity values for species delineation (<98.65% [35]) of the six strains relative to the type strains of other AAB genera suggest that they represent a yet undescribed species and possibly a new genus of the family *Acetobacteraceae*.

A phylogenetic tree based on the 16S rRNA gene sequence of the six isolates and their closest relative type strains was constructed by using the neighbour-joining and maximum-likelihood method in MEGAX (v10.1.8) with 1000 resampling bootstrap after aligning the sequences with the sina software [36,37]. The six strains cluster with uncultivated bacteria, including *Acetobacteraceae* sp., *Asaia* spp. and *Ca*. K. diaphorinae, on a branch of the phylogenetic tree well separated from the other cultivated types of AAB genera (Fig. S2). This cluster relates to the *Asaia* clade and the more recently described *Swaminathania* and *Kozakia* genera, whose members were isolated from several sources, including holometabolous insects, fruits, flowers and fermented food [3,14, 38,40].

Although the identity values of these 16S rRNA gene sequences against the closest relative species were above the threshold of taxon boundaries to demarcate a novel genus, which is generally considered <95% [41], the long branches observed for this new cluster in the phylogenetic tree (Fig. S2) suggest a significant divergence from the ancestor sequence, which probably reflects a rapid evolution to adapt to the insect host. Moreover, the fact that our strains and the *Ca*. K. diaphorinae are associated with hemipterans, which diverged from the other insect lineage 300 million years ago [26], makes us hypothesize that such isolates evolved uniquely, forming a novel genus.

## Phylogenomics and genome relatedness

Since the six isolates from *E. variegatus* showed identical sequences for the 16S rRNA gene and 16S–23S rRNA gene internal transcribed spacer [42], as well as clonality (i.e. ‘sister strains’, see the Enterobacterial Repetitive Intergenic Consensus PCR in Fig. S3), we further analysed the genome of EV16P^T^, here proposed as type strain of such clade, to investigate in detail its phylogenetic placement. The genomic DNA was extracted using Maxwell RSC Automated Nucleic Acid Purification and Maxwell RSC Cultured Cells DNA kits (Promega) starting from bacterial cultures grown at 30 °C in GYCE for 72 h. The extracted DNA was quantified using Qubit dsDNA assay kits with high sensitivity (Thermo-Fischer Scientific), and its quality was checked using a Bioanalyzer 2100 (Agilent) before being sequenced using a PacBio RS2 sequencer (Pacific Biosciences) at the Bioscience Core Lab at King Abdullah University of Science and Technology (KAUST, Saudi Arabia). Whole-genome assemblies were obtained using the HGAP.3 workflow (v2.3.0) [43]. The contigs were assembled into scaffolds with the RagTag (v2.10) software [44] using the genome of *Ca*. K. diaphorinae as reference. The Bakta genome annotation pipeline (v1.8.2), eggNOG-mapper (v2.12) and microTrait software (v1.0.0) were used for genome annotations and metabolic pathways’ identification via the KEGG pathway database [45,48], while we used geNomad (v1.7) to determine the presence of plasmids and prophages in the genomes [49]. The prediction of carbon sources used by the strain based on the genome was made using the GapMind software [50]. We further used Anv’io (v8) to compare the different genomes [51]. The general parameters of the genomes are summarized in Table 1 (see also ‘Genome Features’ paragraph).

**Table 1.** Genome characteristics of the strain EV16P^T^ proposed as a novel genus and related genera in the *Acetobacteraceae* family. Strains: 1, EV16P^T^ (this work, JAWJZY000000000); 2, *Asaia bogorensis* 71^T^ (LMG 21650^T^ [65]); 3, *Swaminathania salitolerans* PA51^T^ (LMG 21291^T^ [38]); 4, *Kozakia baliensis* Yo-3^T^ (LMG 21812^T^ [66]); 5, *Candidatus* Kirkpatrickella diaphorinae (CP107052; [9]). *Determined using geNomad [49].

Characteristics	1	2	3	4	5
Size (bp)	2 388 582	3 198 265	2 927 457	3 505 604	2 176 471
Contigs	6	1	9	7	1
DNA G+C (%)	57	59.1	62.85	57.4	57.1
Coding sequence	2330	2749	2483	2845	1857
Plasmid*	1	0	6	0	0
Prophages*	7	3	2	2	2
No. of rRNA genes					
5S	3	5	1	5	3
16S	4	5	2	5	3
23S	3	5	1	5	3
No. of tRNA	54	57	45	57	53

**Fig. 1.**
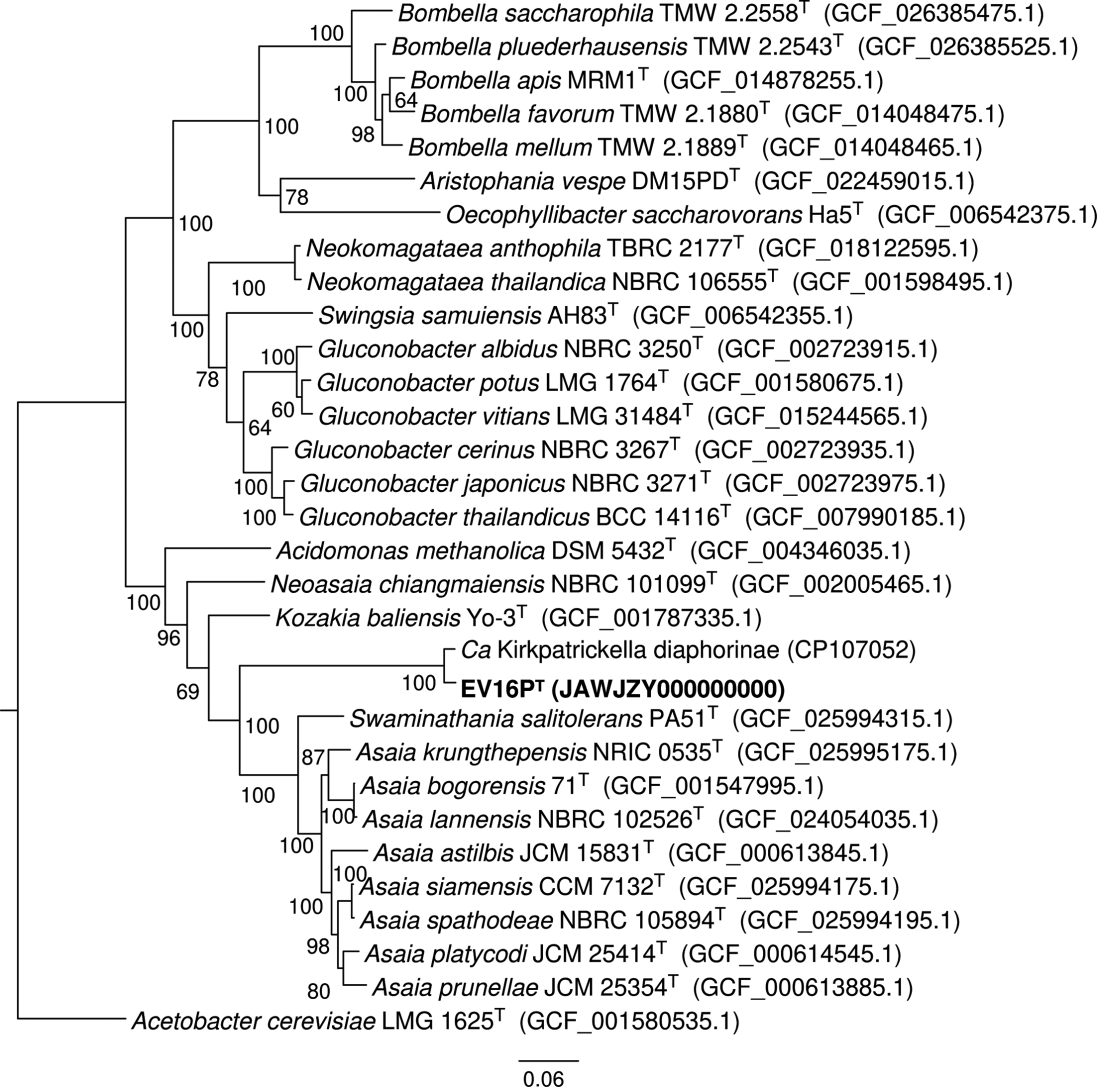
Phylogenetic tree with proposed genus (EV16P^T^) and related taxa based on 120 concatenated single-copy genes using the maximum-likelihood method made using the software GTDB-Tk [52]. The NCBI accession numbers are given after the species name. The tree is drawn to scale, with branch lengths measured in the number of substitutions per site. Numbers at nodes indicate bootstrap percentages using 1000 replicates. *Acetobacter cerevisiae* LMG 1625^T^ was used as the outgroup. Bar, 0.06 substitutions per site.

A multilocus sequence analysis and a phylogenetic tree, which included the alignment of 120 concatenated single-copy conserved marker genes of bacterial genomes, were conducted *de novo* using the GTDB-Tk software [52] on the EV16P^T^ isolate and selected type strains within the family *Acetobacteraceae*, including among others *Asaia* spp., *S. salitolerans*, *K. baliensis*, *Bombella* spp. and *Gluconobacter* spp., along with *Ca*. K. diaphorinae (species names and accession numbers in Table S2). The alignment of the concatenated genes confirms that EV16P^T^ and *Ca*. K. diaphorinae cluster in a monophyletic branch originating from the trajectory leading to *Swaminathania* and *Asaia* but positioned after the branch separation leading to *Kozakia* (Fig. 1). The location of the EV16P^T^ branch suggests that it represents an evolutionary intermediate between *Kozakia* and *Swaminathania*. Over the 120 core proteins, the branch length distances to the EV16P^T^ tip are 0.31, 0.32 and 0.33 substitutions per amino acid for *S. salitolerans* PA51^T^, *K. baliensis* Yo-3^T^ and *A. krungthepensis* NRIC 0535^T^, respectively, revealing that our strain is evolutionarily closer to *Swaminathania* than to other validly named taxa.

We further applied several genome distance metrics to compare EV16P^T^ to the type strains of the closest related genera *Asaia* (eight species), *Swaminathania* (1) and *Kozakia* (1), as well as *Ca*. K. diaphorinae (Table S2). First, pairwise average nucleotide identity based on blast (ANIb) was calculated using the JSpeciesWS software with default parameters [53]. The proposed strain exhibits a maximum ANIb value of 68.35% with *A. bogorensis* 71^T^ (Table 2, all pair comparisons in Fig. S4A). The ANIb value between EV16P^T^ and *Ca*. K. diaphorinae is 89.24%, indicating that they are different species in the same genus. The genome-to-genome distance calculator online server was used to calculate *in silico* the estimated digital value of the DNA–DNA hybridization (dDDH) [54,55]. Comparisons with the closest relative type strains show values ranging from 17.7 to 21.7 (Table 2, Fig. S4B). The comparison of genome relatedness metrics at the nucleotide level, i.e. Microbial Species Identifier [56], further shows how among the validly named type species *A. spathodeae* NBRC_105894^T^ and *A. platycodi* JCM_25414^T^ display the highest alignment fraction (AF) value (0.37) and gANI (71.3%), respectively, but *S. salitolerans* PA51^T^ has the highest combination of the two parameters (gANI=70.9% and AF=0.4; Fig. S5A). Considering that the standard criteria to delineate a new bacterial species are 95–96 and 70% for ANIb and dDDH, respectively [53,55, 57,60], our data support the identification of the EV16P^T^ as a novel species. However, since the uniqueness of the association with hemipterans of our strain and *Ca*. K. diaphorinae and their cluster in a new branch in the phylogenomic tree (Fig. 1), we evaluated the possibility that EV16P^T^ forms a new genus by calculating genome relatedness metrics at the protein level, including average amino acid identity (AAI) [54,61, 62] and percentage of conserved proteins (POCP) (Table 2, Fig. S4C, D) [63,64]. The highest AAI value with type strains is 65.9% and corresponds to *A. platycodi* JCM_25414^T^, while the AAI value between EV16P^T^ and *Ca*. K. diaphorinae is 93.5%, confirming that these two bacteria are different species but make part of the same genus. The highest POCP value is calculated by comparing EV16P^T^ with *S. salitolerans* PA54^T^ (61.2%), while the lowest value is with *Kozakia baliensis* DSM 14400^T^ (51.5%). The relationship between these two parameters reveals high genus demarcation at the protein level compared to those based on nucleotide sequences, with *S. salitolerans* PA54^T^ as the closest strain to EV16P^T^ (Fig. S5B).

**Table 2.** Average nucleotide identity via blast (ANIb), aligned percentage (aligned), *in silico* dDDH, AAI, POCPs, as well as the Microbial Species Identifier (MiSI) composed of gANI and AF of EV16P^T^ with type strains of selected closest genera within the *Acetobacteraceae* family (Figs 1 and S1); *Ca*. Kirkpatrickella diaphorinae [9] was also included. Information related to the selected representatives is reported in Table S2. All values are expressed as percentage (%). Matrices with all pair comparisons are provided for each parameter in Fig. S4. The boundary values proposed for species delineation are ANIb and AAI of 95% and dDDH of 70% [53,57], while the genus delineation values are arbitrarily set as POCP of 50% and AAI of 65–72% [58,63].

Closest relative species	ANIb	Aligned	dDDH	AAI	POCP	gANI/AF
*Asaia astilbis* JCM 15831^T^	67.5	31.4	17.7	65.5	54.3	71.0/0.3
*Asaia bogorensis* 71^T^	68.4	30.9	20.8	65.6	57.8	70.9/0.4
*Asaia krungthepensis* NRIC 0535^T^	67.9	29.0	19.4	65.6	55.4	70.9/0.4
*Asaia lannensis* NBRC 102526^T^	68.1	30.9	19.5	65.5	57.4	71.0/0.4
*Asaia platycodi* JCM 25414^T^	67.7	32.1	18.3	65.9	53.8	71.3/0.3
*Asaia prunellae* JCM 25354^T^	67.4	30.3	19.8	65.8	54.4	70.9/0.3
*Asaia siamensis* NRIC 0323^T^	67.8	30.3	19.6	65.7	56.2	70.9/0.4
*Asaia spathodeae* NBRC 105894^T^	67.8	29.2	18.9	65.5	54.5	70.7/0.4
*Kozakia baliensis* Yo-3^T^	67.6	28.8	21.7	65.6	51.5	70.9/0.3
*Swaminathania salitolerans* PA54^T^	67.7	34.2	18.8	65.7	61.2	70.9/0.4
*Ca* Kirkpatrickella diaphorinae	89.2	94.4	37.9	93.5	89.1	89.9/0.9

Overall, the low AAI (<66%) and POCP (<62%) values at the boundary of the genus threshold, along with the fact that the genome relatedness thresholds are not fixed or universally agreed upon, strongly support that EV16P^T^ should be assigned to a new species within a novel genus in the family *Acetobacteraceae*, for which *S. euscelidii* gen. nov., sp. nov. name is proposed. It is the cultivable representative of the genus *Sorlinia* to which the uncultivated *Ca*. K diaphorinae is highly related.

## Genome features

**Fig. 2.**
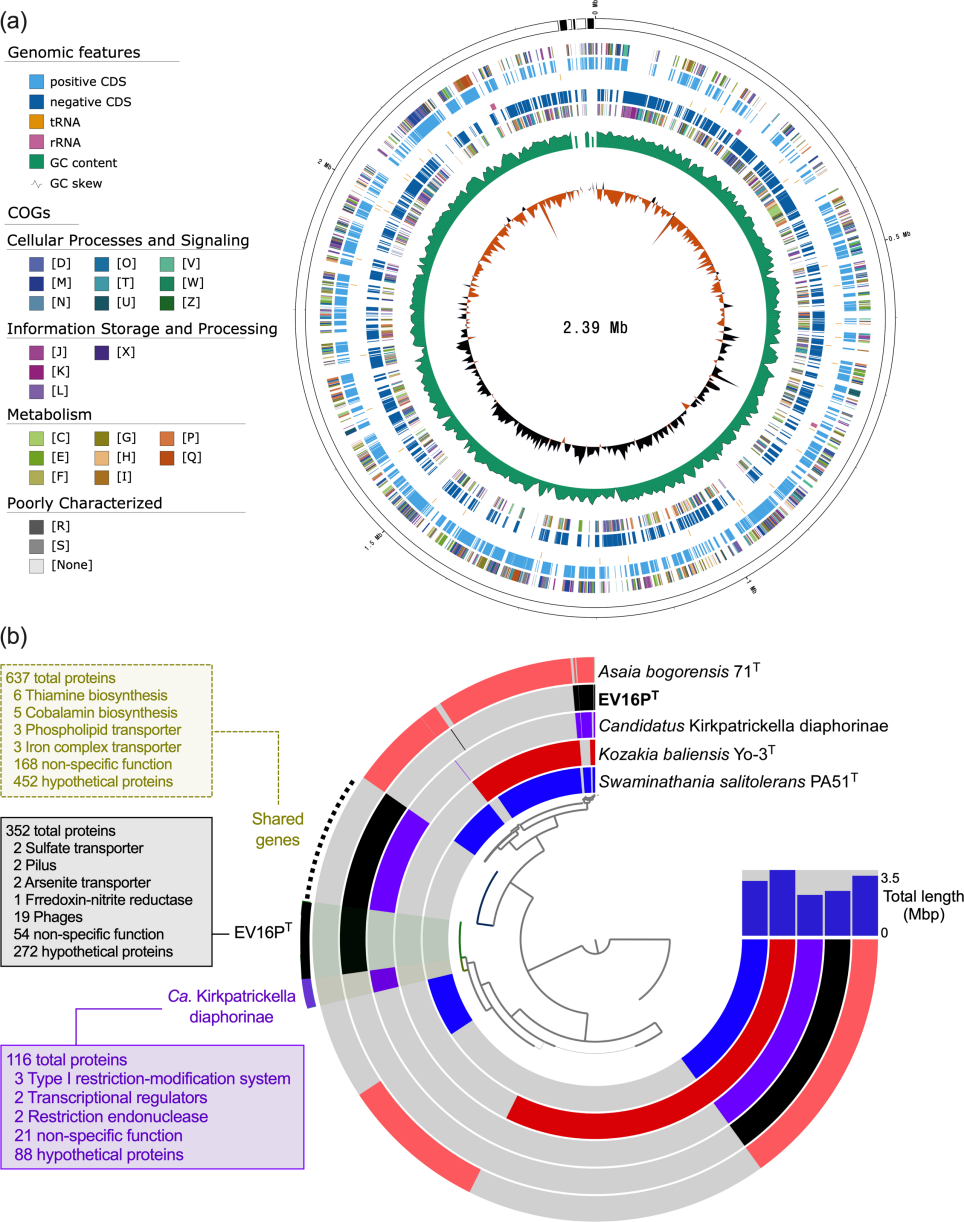
(**a**) Genome map of EV16P^T^, with the following tracks displayed from outside to the inside: Contigs; COGs on the forward strand; CDS, tRNAs and rRNAs on the forward strand; CDS, tRNAs and rRNAs on the reverse strand; COGs on the reverse strand; G+C content; G+C skew [80]. (**b**) Anvi’o pangenome [51] visualization of strain EV16P^T^ and its three closest strains, *Asaia bogorensis* 71^T^ [65], *Swaminathania salitolerans* PA51^T^ [38], *Kozakia baliensis* Yo-3^T^ [66] and *Ca*. Kirkpatrickella diaphorinae [9]. A hierarchical clustering based on the presence (colored portions) and absence (grey portions) of gene clusters (*n* = 5401) across genomes determined the genome ordering in the display; gene clusters detected in each strain are represented by distinct colours: *A. bogorensis* 71^T^ in pink, EV16P^T^ in black, *Ca*. Kirkpatrickella diaphorinae in purple, *K. baliensis* Yo-3^T^ in red and *S. salitolerans* PA51^T^ in blue. Gene clusters unique to strain EV16P^T^ (352) and *Ca*. Kirkpatrickella diaphorinae (116) are highlighted by external black and purple lines, respectively, while gene clusters shared by the two strains (637) are indicated by a dashed black line.

The genome of EV16P^T^ spans a length of 2.389 Mb, exhibiting a G+C% of 57 mol% (Table 1, Fig. 2a). The assembly resulted in 6 contigs, encompassing 2399 genes, of which 2330 were identified as protein-coding genes (CDS). In comparison to the genomes of *A. bogorensis* 71^T^ [65], *S. salitolerans* PA51^T^ [38] and *K. baliensis* Yo-3^T^ [66] – all bacteria that have been isolated from fermented food or plant material – the genome of EV16P^T^ showed a substantial size reduction (0.809, 0.539 and 1.117 Mbp, respectively; Table 1, Fig. 2b). This reduction supports the evolutionary process of a symbiotic-like relationship in which the bacterial genomes undergo a reduction in size over time [67], as also documented for the uncultivated symbiont of the hemipteran *D. citri*, *Ca*. K. diaphorinae (metagenome-assembled genome 2.176 Mbp, as listed in Table 1).

Key genomic features of EV16P^T^ include the presence of 10 rRNA genes (three 5S, four 16S and three 23S rRNA genes), 54 copies of tRNA, 1 annotated plasmid and the identification of 7 integrated prophages (Table 1, Fig. 2a). Notably, the four 16S rRNA genes have sequence similarity of 100%, with three of them found within operons. The 7.7 kbp plasmid comprises 11 CDS, including noteworthy elements such as the outer membrane lipoprotein (NodT), a Ca^2+^-binding protein, an RTX toxin (Repeats in ToXin)-related protein and a glycosyl hydrolase (GH15). The seven predicted prophages predominantly contain viral proteins, with notable inclusions of two glycosyl hydrolases (GH108), a BrnA antitoxin protein, an antitoxin HigA-1 protein, a lysozyme gene and a replicative DNA helicase. EV16P^T^ is an aerobic (via the Cytochrome c oxidase *cyoABCDE*) mixotroph capable of synthesizing all amino acids, with genes involved in assimilatory sulphate reduction but lacking the capacity to biosynthesize nitrogen compounds. The strain lacks mobility genes governing chemotaxis and flagella but possesses genes involved in pili formation (*cpaACF*, DOFOFD_03540-50, DOFOFD_03565). Interestingly, EV16P^T^ does not have several transporters found in the AAB we selected as closely related strains, such as those for carbohydrate acid, polysaccharide and nucleoside. However, it does possess key regulatory systems for nitrogen compounds (*ntrY/ntrX*; DOFOFD_01835-45), phosphate transport (*pstABCS*; DOFOFD_00600-30), lipoprotein transport (*lolCDE*; DOFOFD_11015-20), lipopolysaccharides transport (*lptBFG*; DOFOFD_06240-50, DOFOFD_10815-20, DOFOFD_07020), haem transport (*ccmABC* excluding *ccmD*; DOFOFD_05470-75, DOFOFD_06305) and phospholipid transport (*mlCDEF* excluding *mlB*; DOFOFD_10665-75, DOFOFD_03940-45, DOFOFD_01105, DOFOFD_07810, DOFOFD_00795, DOFOFD_04945). Several genes related to exopolysaccharide biosynthesis (*epsBCDEFHJMN*) are also present, with a few omissions (*epsAGIKLO*). Additional polysaccharide-related functions include the degradation of cellulose, chitin and xylan. Differently from its closely related strains, EV16P^T^ possesses only the genes encoding the fermentation of pyruvate to short-chain fatty acids but not to alcohols, and it lacks genes involved in carotene biosynthesis (e.g. neurosporene and lycopene), mRNA chaperone system and phage-shock system.

Further analysis focused on metabolic pathways linked to symbiosis with insects [68,69] was conducted. The functional annotation of genes involved in the major metabolic pathways of (and not limited to) insect-associated AAB, including the ethanol oxidation respiratory chain pathway, the TCA pathway, the pyruvate metabolic pathway and the pentose phosphate pathway, was performed using the eggNOG-mapper software (v2.12) and microTrait software (v1.0.0) [45,48]. While EV16P^T^ possesses the pyruvate metabolic pathway and the pentose phosphate pathway, it does not encode for the ethanol oxidation pathway (*adhAB* and *aldh*) and lacks a gene involved in the TCA pathway (malate dehydrogenase, *mqo*) but possesses the 14 others. Based on the metabolic prediction, EV16P^T^ uses as C-sources glucose, glycerol, cellobiose, proline, aspartate, fumarate, l-malate, 2-oxoglutarate, succinate, threonine, acetate and asparagine. It also encodes for the *aatA* gene (DOFOFD_11430), an ATP-binding cassette transporter that influences acid resistance and may act as an efflux pump for acetic acid [70] in case the latest is produced by bacterial cells. The two-component system *chvI*/*chvG* (DOFOFD_03150-55), which has been shown to have a direct influence on bacterial physiology, including host interactions [71], and on regulating acid-inducible genes [72], is also present in the genome of EV16P^T^. Among the other bacterial functions linked to the symbiosis/association with insects [69], the Sec-SRP (DOFOFD_00235, DOFOFD_04705, DOFOFD_06590-95, DOFOFD_08025, DOFOFD_09495, DOFOFD_11415-20) and Tat (DOFOFD_02550, DOFOFD_10475-80) secretion systems are present in EV16P^T^, while genes associated with acetoin and 2,3-butanediol biosynthesis are absent. Notably, gene annotation does not reveal gene encoding for cellulose production (cellulose synthase, *BcsA*), as observed in other AAB [73].

To understand the possible genetic clues for the successful cultivation of EV16P^T^, we analysed the portion of the genome we detected only in our strain and not in *Ca*. K diaphorinae symbiont. It consists of 352 genes, for which only a limited number of CDS has been assigned a function (Fig. 2b). While most of those genes are either linked to phages (19) or hypothetical proteins (272), minor differences in functional genes were observed, including pili genes (described above) and genes coding for a ferredoxin-nitrite reductase (*nirA*, DOFOFD_02065), transcriptional regulators involved in the resistance to arsenite (*arsR*, DOFOFD_01500, DOFOFD_07350) or sulphate transporters (DOFOFD_01530, DOFOFD_07320). Although we did not find any specific genes related to ‘cultivability clues’, the possibility that the isolation of EV16P^T^ is encoded within this portion of the genome cannot be ruled out. At the same time, we cannot exclude those differences in cultivability stem from the use of distinct cultivation strategies and conditions, even though in both cases a medium with d-sorbitol as the primary carbon source was used for the initial enrichment.

## Chemotaxonomy

The type strains *A. bogorensis* 71^T^ [31], *Swaminathania salitolerans* PA51^T^ [38] and *Kozakia baliensis* Yo-3^T^ [39], all species related to EV16P^T^, were obtained from the Belgian Coordinated Collections of Microorganisms (BCCM)/LMG bacteria catalogue (LMG 21650^T^, LMG 21291^T^ and LMG 21812^T^, respectively) and grown following the recommended culture conditions. Bacterial biomass from EV16P^T^ and the selected reference strains grown in GYCE media for 3 days at 30 °C were collected, and analyses of respiratory quinones, polar lipids and cellular fatty acids (CFAs) were carried out by the DSMZ services, Leibniz-Institute DSMZ (Deutsche Sammlung von Mikroorganismen und Zellkulturen GmbH, Braunschweig, Germany).

As for all the AAB type strains closely related to EV16P^T^ analysed here, the respiratory quinones are all ubiquinones (Q), with Q-10 as the major component (Table S3). The CFA profiles of strain EV16P^T^ and related bacteria showed distinct features for genera differentiation, but similarities between all species are evident (Table 3, Fig. S6). EV16P^T^ is clearly distinguished by the lowest proportion of monounsaturated C_18 : 1_* ω7*c and the highest of C_19 : 0_ cyclo *ω*6c compared with the type representative of the selected genera. Notably, our strain has equal distribution between saturated fatty acids (straight-chain and hydroxy) and monounsaturated, both with several components. The polar lipid profile of EV16P^T^ comprises diphosphatidylglycerol, phosphatidylethanolamine, phosphatidylcholine and unidentified aminophospholipid (*n*=1), glycophospholipid (1), aminolipids (3) and lipids (3). Its polar lipid profile is similar to those of *S. salitolerans* and *K. baliensis* in having diphosphatidylglycerol, phosphatidylethanolamine, phosphatidylcholine but not for the lipidic portions (Fig. S7).

**Table 3.** Cellular fatty acid contents of proposed and the reference type strains. Strains: 1, EV16P^T^ (this work); 2, *Asaia bogorensis* 71^T^ (LMG 21650^T^); 3, *Swaminathania salitolerans* PA51^T^ (LMG 21291^T^); 4, *Kozakia baliensis* Yo-3^T^ (LMG 21812^T^). Data were obtained in this study (Fig. S6). The prevalent fatty acid components (>10%) in strain EV16P^T^ are highlighted in bold. Fatty acids accounting for less than 1% in all the strains are not reported; TR, trace (less than 1.0%); –, not detected.

Fatty acids		1	2	3	4
Saturated	C_14 : 0_	tr	tr	1.2	1.3
	C_14 : 0_ 2-OH	tr	5.1	5.6	2.1
	C_14 : 0_ 3-OH	2.0	1.3	1.1	tr
	C_16 : 0_	**19.3**	**12.9**	**14.1**	**18.8**
	C_16 : 0_ 2-OH	3.6	–	5.3	3.2
	C_16 : 0_ 3-OH	3.2	–	tr	–
	C_18 : 0_	4.4	2.2	1.7	3.3
	C_19 : 0_ cyclo *ω*6c	**22.3**	tr	5.7	3.5
Monounsaturated	C_13 : 1_	2.5	–	–	–
	C_17:l_ *ω*6c	1.0	tr	–	–
	C_18 : 1_* ω*7c	**26.1**	**68.1**	**55.5**	**64.1**
	C_18 : 1_* ω*6c	1.1	–	–	–
	C_18 : 1_ DMA	1.0	–	–	–
	C_19 : 1_ 2-OH	**11.5**	6.8	7.7	–

## Physiology

All experiments were performed on both proposed and described species. Unless otherwise stated, EV16P^T^ was grown on the GYCE medium, and the type strains were grown on the culture media recommended by BCCM.

Cell morphology was visualized via scanning electron microscopy (Nova Nano and a Quattro FEI SEM) at the Imaging Core Lab at KAUST. ImageJ software was used to determine the dimensions of individual cells. Gram staining was performed following the standard protocol [74]. To evaluate cell motility, 10 µl of liquid culture was inoculated into soft agar [0.3% (w/v) agar] medium and incubated at 30 °C for 72–96 h (adapted from [75]). The formation of pellicles was tested in static cultures at 30 °C for 7 days using potato dextrose broth (PDB) medium [76]. After incubation, cultures were tested for cellulose formation by staining the colonies on the plates with 1% Congo red solution. Growth in microaerophilic or anaerobiosis conditions was tested with CampyGen and AnaeroGen (Thermo Scientific), respectively. Bacteria were incubated at 10, 15, 20, 25, 28, 30, 33, 37 and 40 °C for 5 days to determine temperature tolerance and optimum. The halotolerance and capacity to grow at different pH levels were investigated using the Phenotype Microarray Biolog PM9 and PM10 plates, respectively. Growth on single carbon sources was carried out by inoculating Phenotype Microarray Biolog PM1 and PM2 in the IF0 solution. Bacterial growth was monitored in media containing 1% (w/v) yeast extract with 1, 2, 10, 15, 20 and 30% (w/v) d-glucose, as well as in mannitol agar, glutamate agar, 0.35% acetic acid agar and different concentrations of ethanol (0.5, 1, 2, 3, 4 and 5%, v/v). The capacity to grow on Luria–Bertani (LB) agar and tryptic soy agar (TSB) was also tested. The ability to oxidize ethanol to acetic acid was performed on Carr agar medium containing ethanol as carbon source and bromocresol green as pH indicator [77], as well as in GYCE liquid medium prepared without glucose and calcium carbonate, but with the addition of different concentrations of ethanol (0.5, 1, 2, 3, 4 and 5%, v/v). Catalase activity was determined by adding a drop of 3% (v/v) H_2_O_2_ to whole bacterial cells and oxidase activity by adding a tetramethyl-p-phenylenediamine solution to a cell suspension. API 50CH, API ZYM and API 20 NE test kits (bioMérieux) were used according to the manufacturer’s recommendations for the additional phenotypic tests.

**Fig. 3.**
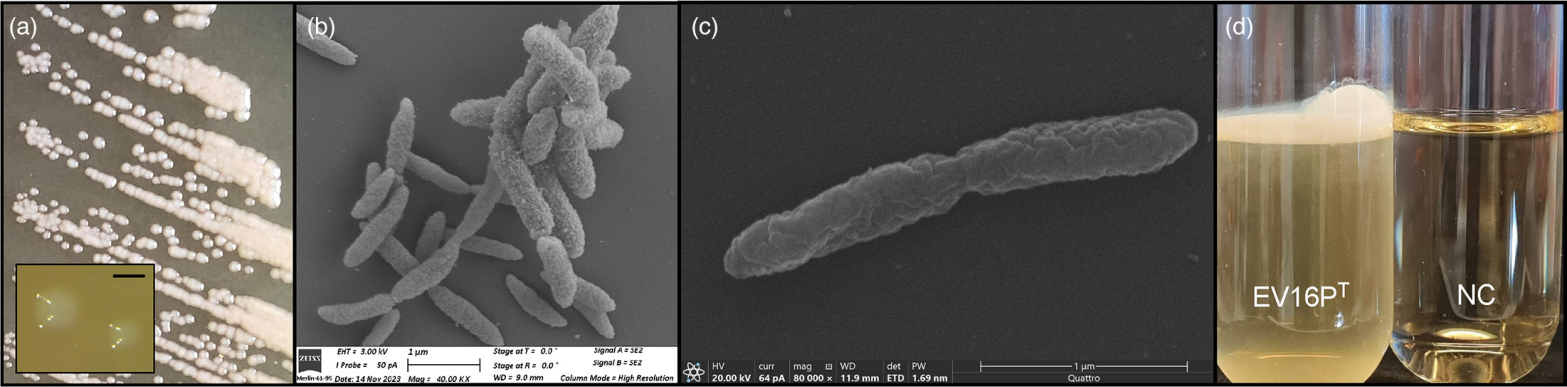
(**a**) Morphology of EV16P^T^ colonies grown on GYCE plates at 3 days and the inset showing colonies observed under the stereomicroscope (bar length, 0.2 mm). (**b and c**) Scanning electron micrographs of EV16P^T^ cells grown on GYCE medium at 30 °C. Bars’ lengths are 1 µm. (**d**) Pellicle formation of EV16P^T^ (*vs* non-inoculated medium, NC) during growth in PDB under static conditions for 7 days.

EV16P^T^ is a Gram-stain-negative, non-sporulating, non-motile, rod-shaped bacterium. When growing on GYCE agar medium at 30 °C for 3 days, the colonies are creamy-beige and circular with 0.3 mm average diameter and entire margins (Fig. 3a). At SEM, the cells appear rugose with 1.4±0.3 µm in length and 0.18±0.06 µm in width (Fig. 3b, c). It is mesophilic (15–33 °C, optimum 30 °C), grows over a wide pH range (4.5–8.0, optimum 6.0–7.0), is halosensitive (0–1% (w/v) NaCl, optimum 0%) and does not grow at high glucose concentrations as the other type strains tested (Table 4). Notably, the growth of all strains tested is inhibited by ethanol concentrations >2%. EV16P^T^ can grow on mannitol and TSB, as well as on media indicated for the cultivation of the selected type strains (BCCM media, M360, M105, M404, M13 and M17), but it does not grow on LB or glutamate. The utilization of the following compounds as a sole source of carbon was positive: glucose, glycerol, arabinose, mannose, aspartate, fumarate and succinate. It is strictly aerobic and can grow under microaerobic conditions. The doubling time under optimum conditions is 6.6 h. After incubation in static conditions for 5 days at 30 °C, the strain forms a thin pellicle on the surface of the liquid medium (Fig. 3d). Even though AAB pellicles mainly comprise exopolysaccharides, among which cellulose could be one of the components [78], the EV16P^T^ strain does not show any evidence of cellulose production. In fact, boiling of the produced pellicles in NaOH resulted in any residual [79], confirming that the EV16P^T^ strain is not able to produce cellulose, as also indicated by genome analysis that does not detect key genes related to cellulose biosynthesis (i.e. *BcsA*).

**Table 4.** Physiological and biochemical features differentiating EV16P^T^ from the selected type strains. 1: EV16P^T^ (this work); 2, *Asaia bogorensis* 71^T^ (LMG 21650^T^); 3, *Swaminathania salitolerans* (LMG 21291^T^); 4, *Kozakia baliensis* Yo-3^T^ (LMG 21812^T^). +, Positive reaction; –, negative reaction; w, weak reaction. All results are from this study. AA, acetic acid.

Physiology	1	2	3	4
Colony colour	Creamy-beige	Beige-pink	Pink	Beige-pink
Pellicle formation in PDB	+	–	–	w
Temperature range (°C)	15–33	15–37	15–37	15–33
Temperature optimum (°C)	30	30–33	30–33	25–37
pH range	4.5–7.0	4.0–10.0	3.5–10.0	3.5–8.0
pH optimum	6.0–7.0	7.0–10.0	5.5–8.0	5.0–8.0
NaCl range (%, w/v)	0–1	0–1 (w)	0–6.5	0
NaCl optimum (%, w/v)	0	0	0–4	0
Glucose range (%, w/v)	1–15	1–30	1–30	1–30
Glucose optimum (%, w/v)	1.5–2	1.5–5	1.5–5	1.5–5
Growth on:				
Mannitol	w	+	+	+
Glutamate	–	+	+	–
EtOH	–	–	w	w
TSB	+	+	+	–
LB	–	w	w	w
Catalase activity	+	+	+	+
Oxidase activity	–	–	–	–
API 20NE (**Fig. S8**), activity of:				
Aesculin	+	w	+	w
API ZYM (**Fig. S9**), activity of:				
Esterase (C4)	–	+	+	+
Leucine arylamidase	+	+	+	w
Valine arylamidase	–	+	+	+
Trypsin	+	–	–	–
Acid phosphatase	+	+	+	–
Naphthol-AS-BI-phosphohydrolase	+	+	+	w
⍺-Glucosidase	–	–	+	–
*β*-Glucosidase	+	–	–	–
*N*-acetyl-*β*-glucosaminidase	–	–	+	–
API 50CH (**Fig. S10**), acid production from:				
Glycerol	+	+	+	+
d-Xylose	+	+	+	+
d-Glucose	+	+	+	+
d-Mannose	+	+	+	–
Inositol	+	+	w	–
Aesculin	+	–	+	–
Lyxose	+	–	w	–
5-Keto-gluconate	+	+	–	w
AA production (ABEM/GYC agar)	–/–	+/w	+/+	+/+
Oxidation of EtOH into AA (Carr agar)	–	–	+	+

As described for most AAB, EV16P^T^ has catalase-positive and oxidase-negative phenotypes. According to the API 20 NE test strip, the species is positive for aesculin hydrolysis only (Fig. S8), while the API ZYM test strip shows positive activity for leucine arylamidase, trypsin, acid phosphatase, naphthol-AS-BI-phosphohydrolase and *β*-glucosidase (Fig. S9). The EV16P^T^ strain produces acids fermenting several carbohydrates, including glycerol, d-xylose, d-glucose, d-mannose, inositol, aesculin, lyxose and 5-keto-gluconate (API 50 CH test strip, Fig. S10). Still, it does not oxidize ethanol to acetic acid. Notably, the type strains of related genera, especially *A. bogorensis* 71^T^ (LMG 21650^T^) and *S. salitolerans* LMG 21291^T^, have a wider range of enzymatic activities and fermenting abilities than EV16P^T^ (Table 4, Figs S8–S10). More specific phenotypes, including key biochemical characteristics of the proposed strain and related species, are presented in Table 4.

Based on the genomic, phenotypic, physiological and chemotaxonomic characteristics and the results of the phylogenetic analyses discussed above, strain EV16P^T^ appears to represent a novel species in a new genus within the *Acetobacteraceae* family, for which we propose the name *S. euscelidii* gen. nov. sp. nov.

## Description of *Sorlinia* gen. nov.

*Sorlinia* (Sor.li’ni.a. N.L. fem. n. *Sorlinia*, subjective name derived from the surname Sorlini, named in honour of Prof. Claudia Sorlini at the University of Milan, Italy).

Cells are Gram-negative, aerobic, non-motile, mesophilic rods that are catalase-positive and oxidase-negative. Ubiquinone Q-10 is the primary respiratory quinone, and dominant CFAs (>10%) are C_18:l_*ω*7c, C_19 : 0_ cyclo *ω*6c, C_16 : 0_ and C_19 : 1_ 2OH. Polar lipids consist of diphosphatidylglycerol, phosphatidylethanolamine and phosphatidylcholine, along with one unidentified aminophospholipid, one unidentified glycophospholipid, three unidentified aminolipids and three unidentified lipids.

The genus *Sorlinia* belongs to the family *Acetobacteraceae*. The type species is *S. euscelidii*.

## Description of *S. euscelidii* sp. nov.

*S. euscelidii* sp. nov. (eu.sce.li’di.i. N.L. gen. n. *euscelidii*, of the genus *Euscelidius*, an insect genus of the *Hemiptera* order from which the bacterium was isolated).

The species has the features of the genus, with the following additions. Cells are rod-shaped, 1.4±0.3 µm long and 0.18±0.06 µm wide; colonies are 0.3 mm in diameter, cloudy inside and transparent towards the edges with creamy clear surfaces when grown on GYCE agar plates for 72 h. Growth occurs at 15–33 °C (optimum 30 °C), pH 4.5–8.0 (6.0–7.0), with 0–1% (w/v) NaCl (0%) and with 1–15% (w/v) glucose (2%). It is strictly aerobic and can grow under microanoxic conditions. No growth occurs on glutamate agar or lysogeny broth agar, while weak growth is observed on TSB and mannitol agar. In liquid culture, pellicles are formed, but no cellulose. The utilization of the following compounds as a sole source of carbon is positive: glucose, glycerol, arabinose, mannose, aspartate, fumarate and succinate. Acid is produced (API 50CHB/E) from glycerol, d-xylose, d-glucose, d-mannose, inositol, aesculin, lyxose and 5-keto-gluconate, but ethanol is not oxidized to acetic acid. The enzyme activities of leucine arylamidase, trypsin, acid phosphatase, naphthol-AS-BI-phosphohydrolase and *β*-glucosidase are detected with the API ZYM test system.

The type strain, EV16P^T^ (= KCTC 8296^T^, = DSM 117028^T^), was isolated from *E. variegatus* reared at the University of Turin, Italy. The genome size is 2 388 582 bp, and the DNA G+C content is 57.0%. The NCBI accession number of the genome and the 16S rRNA gene sequence are JAWJZY000000000 and OR678267, respectively.

## supplementary material

10.1099/ijsem.0.006544 Uncited Fig. S1.

## Abbreviations

AA: acetic acid
AAB: acetic acid bacteria
AF: alignment fraction
ANIb: average nucleotide identity based on BLAST
CDS: protein-coding sequences
dDDH: *in silico* digital DNA–DNA hybridization
GGDC: genome-to-genome distance calculator
GYCE: glucose-yeast-CaCO_3_-ethanol
LPSN: List of Prokaryotic names with Standing in Nomenclature
MLSA: multilocus sequence analysis
NCBI: National Center for Biotechnology Information
POCP: percentage of conserved proteins
rRNA: ribosomal ribonucleic acid
