# Supplementary material for: Sorlinia euscelidii gen. nov., sp. nov., a novel acetic acid bacterium isolated from the leafhopper Euscelidius variegatus (Hemiptera: Cicadellidae)
Source: Int J Syst Evol Microbiol. 2024 Oct 21;74(10):006544. doi: 10.1099/ijsem.0.006544 (PMC11493185; doi:10.1099/ijsem.0.006544)
Supplement: Uncited Fig. S1. [file ijsem-74-06544-s001.pdf]

SUPPLEMENTARY MATERIAL of

***Sorlinia euscelidii* gen. nov., sp. nov., a novel acetic acid bacterium isolated from the leafhopper *Euscelidius variegatus* (*Hemiptera*: *Cicadellidae*)**

Ramona Marasco[1,†], Grégoire Michoud[1,†], Kholoud A. Seferji[1], Elena Gonella[2], Elisa Garuglieri[1], Eleonora Rolli[3], Alberto Alma[2], Francesca Mapelli[3], Sara Borin[3], Daniele Daffonchio[1*], Elena Crotti[3*]

[1]Biological and Environmental Sciences and Engineering Division (BESE), King Abdullah University of Science and Technology (KAUST), Thuwal, Saudi Arabia
[2]Department of Agricultural, Forest, and Food Sciences (DISAFA), University of Torino, Turin, Italy
[3]Department of Food, Environmental and Nutritional Sciences (DeFENS), University of Milan, Milan, Italy

***Correspondence:** Elena Crotti, elena.crotti@unimi.it; Daniele Daffonchio, daniele.daffonchio@kaust.edu.sa

[†]These authors contributed equally to this work

Supplementary Table 1. List of media used in isolation attempts of AAB from *Euscelidius variegatus* and *Scaphoideus titanus*. In bold is indicated the enrichment culture from which we isolated the six strains reported in this work.

Insect	Medium*	Method	Plating following enrichment	Reference
E. variegatus	TA1 (1% glucose, 0.5% ethanol, 0.3% acetic acid, 1.5% peptone, 0.8% yeast extract; pH 3.5)	Enrichment	MA (1% glucose, 1% glycerol, 1% bactopeptone, 0.5% yeast extract, 0.7% $CaCO_3$, 1% ethanol, 1.5% agar, pH 6.8)	[1]
	YE (2% yeast extract, 2% ethanol, 1% acetic acid, pH 6)	Enrichment	MA (1% glucose, 1% glycerol, 1% bactopeptone, 0.5% yeast extract, 0.7% $CaCO_3$, 1% ethanol, 1.5% agar, pH 6.8)	[1]
	**ABEM (2% sorbitol, 0.5% peptone, 0.3 % yeast extract, pH 3.5)**	**Enrichment**	**GYCE (2% glucose, 0.5% ethanol, 0.8% yeast extract, 0.7% $CaCO_3$, 1.2% agar, pH 7)**	[1]
	MAN plates (2.5% mannitol, 0.3% peptone, 0.5% yeast extract, pH 7, agar 15 g/L)	Direct plating	-	[1]
S. titanus	ABEM (2% sorbitol, 0.5% peptone, 0.3% yeast extract, pH 3.5 and pH4)	Enrichment	GYCE (2% glucose, 0.5% ethanol, 0.8% yeast extract, 0.7% CaCO3, 1.2% agar, pH 7)	[2]
	Sucrose-medium (2% sucrose, 0.3% peptone, 0.3% yeast extract, pH 3.5)	Enrichment	MA (1% glucose, 1% glycerol, 1% bactopeptone, 0.5% yeast extract, 0.7% $CaCO_3$, 1% ethanol, 1.5% agar, pH 6.8)	[2]
	Glucose-medium (1% glucose, 1.5% peptone, 0.8 % yeast extract, pH 3.5 and pH4)	Enrichment	MA (1% glucose, 1% glycerol, 1% bactopeptone, 0.5% yeast extract, 0.7% $CaCO_3$, 1% ethanol, 1.5% agar, pH 6.8)	[2]
	Mannitol-medium (1% mannitol, 0.5% peptone, 0.3% yeast extract, pH 3.5)	Enrichment	MA (1% glucose, 1% glycerol, 1% bactopeptone, 0.5% yeast extract, 0.7% $CaCO_3$, 1% ethanol, 1.5% agar, pH 6.8)	[2]
	Mix-medium (1%glucose, 1% ethanol, 1%mannitol, 0.2% yeast extract, 0.2% peptone, pH 4.5)	Enrichment	MA (1% glucose, 1% glycerol, 1% bactopeptone, 0.5% yeast extract, 0.7% $CaCO_3$, 1% ethanol, 1.5% agar, pH 6.8)	[2]
	TA1 (1% glucose, 0.5% ethanol, 0.3% acetic acid, 1.5% peptone, 0.8% yeast extract; pH 3.5)	Enrichment	MA (1% glucose, 1% glycerol, 1% bactopeptone, 0.5% yeast extract, 0.7% $CaCO_3$, 1% ethanol, 1.5% agar, pH 6.8)	[2]

*All media were added with100 µg/ml cycloheximide and incubated at 30 °C under aerobic conditions. Enrichment media were incubated with shaking.

Supplementary Table 2. List of type strains of species of genera and *Candidatus* (*Ca.*) Kirkpatrickella diaphorinae selected as references for comparison analyses. N.A., not available. The type strains selected for biochemical and physiological characterisation and comparison are in bold.

List n.	Species	Type strain	Genome accession number	Reference
1	*Acetobacter cerevisiae*	LMG 1625	GCF_001580535.1	[3]
2	*Acidomonas methanolica*	DSM 5432	GCF_004346035.1	[4]
3	*Aristophania vespe*	DM15PD	GCF_022459015.1	[5]
4	*Asaia astilbis*	JCM 15831	GCF_000613845.1	[6]
**5**	***Asaia bogorensis***	**71**	**GCF_019823045.1**	[7, 8]
6	*Asaia krungthepensis*	NRIC 535	GCF_025995175.1	[9]
7	*Asaia lannensis*	NBRC 102526	GCF_024054035.1	[10]
8	*Asaia platycodi*	JCM 25414	GCF_000614545.1	[6]
9	*Asaia prunellae*	JCM 25354	GCF_000613885.1	[6]
10	*Asaia siamensis*	CCM 7132	GCF_014635085.1	[11]
11	*Asaia spathodeae*	NBRC 105894	GCF_025994195.1	[12]
12	*Bombella apis*	MRM1	GCF_014878255.1	[13]
13	*Bombella favorum*	TMW 2.1880	GCF_014048475.1	[14]
14	*Bombella mellum*	TMW 2.1889	GCF_014048465.1	[14]
15	*Bombella pluederhausensis*	TMW 2.2543	GCF_026385525.1	[15]
16	*Bombella saccharophila*	TMW 2.2558	GCF_026385475.1	[15]
17	*Gluconobacter albidus*	NBRC 3250	GCF_002723915.1	[16]
18	*Gluconobacter cerinus*	NBRC 3267	GCF_002723935.1	[17]
19	*Gluconobacter japonicus*	NBRC 3271	GCF_002723975.1	[18]
20	*Gluconobacter potus*	LMG 1764	GCF_001580675.1	[19]
21	*Gluconobacter thailandicus*	BCC 14116	GCF_007990185.1	[20]
22	*Gluconobacter vitians*	LMG 31484	GCF_015244565.1	[19]
**23**	***Kozakia baliensis***	**Yo-3**	**GCF_001787335.1**	[21, 22]
24	*Neoasaia chiangmaiensis*	NBRC 101099	GCF_002005465.1	[23]
25	*Neokomagataea anthophila*	TBRC 2177	GCF_018122595.1	[24]
26	*Neokomagataea thailandica*	NBRC 106555	GCF_001598495.1	[25]
27	*Oecophyllibacter saccharovorans*	Ha5	GCF_006542375.1	[26]
**28**	***Swaminathania salitolerans***	**PA51**	**GCF_007988945.1**	[27]
29	*Swingsia samuiensis*	AH83	GCF_006542355.1	[28]
30	*Ca.* Kirkpatrickella diaphorinae	N.A.	CP107052.1	[29]

Supplementary Table 3. The detected respiratory quinones were all ubiquinones (Q). Percentages are reported below for EV16P^T and the type strains of selected species of related genera.

Bacterial strain	Respiratory quinones (%)			
	**Q-8**	**Q-9**	**Q10**	**Q11**
EV16P^T		2.8	97.2	
Kozakia baliensis		1.6	98.4	
Asaia bogorensis	0.2	2.1	96.2	1.5
Swaminathania salitolerans	0.5	4.6	94.9	

Supplementary Figure 1. Results of Basic Local Alignment Search Tool (BLAST) alignment of EV16P^T against (**A**) standard databases (nr etc.) and (**B**) rRNA/ITS databases with type strains. Sequence alignment, 5th December 2023. The 16S rRNA gene sequences obtained from the six isolates in this work have 100% identity.

(**A**) Standard databases

Description	Scientific Name	Max Score	Total Score	Query Cover	E value	Per. Ident	Acc. Len	Accession
Uncultured Asaia sp. clone TF.6-8 16S ribosomal RNA gene, partial sequence	uncultured Asaia sp.	2444	2444	100%	0.0	100.00%	1432	MN099438.1
Uncultured Acetobacteraceae bacterium clone MYCCUN1 16S ribosomal RNA gene, partial sequence	uncultured Acetobacte...	2444	2444	100%	0.0	100.00%	1486	OQ100069.1
Uncultured Acetobacteraceae bacterium clone ZOPTEN1 16S ribosomal RNA gene, partial sequence	uncultured Acetobacte...	2444	2444	100%	0.0	100.00%	1486	OQ100068.1
Uncultured Acetobacteraceae bacterium clone PHASUB1 16S ribosomal RNA gene, partial sequence	uncultured Acetobacte...	2427	2427	100%	0.0	99.77%	1486	OQ100071.1
Candidatus Kirkpatrickella diaphorinae isolate CADCRV1 chromosome, complete genome	Candidatus Kirkpatrick...	2422	7266	100%	0.0	99.70%	2176471	CP107052.1
Acetobacteraceae bacterium (ex Diaphorina citri) clone CADcRV01 16S ribosomal RNA gene, partial sequ...	Candidatus Kirkpatrick...	2422	2422	100%	0.0	99.70%	1496	OP600170.1
Uncultured Acetobacteraceae bacterium clone TRYOCC1 16S ribosomal RNA gene, partial sequence	uncultured Acetobacte...	2416	2416	100%	0.0	99.62%	1486	OQ100067.1
Uncultured Asaia sp. clone wbph-rice 16S ribosomal RNA gene, partial sequence	uncultured Asaia sp.	2399	2399	100%	0.0	99.40%	1452	MN094402.1
Uncultured bacterium clone Asaia sp. 16S ribosomal RNA gene, partial sequence	uncultured bacterium	2399	2399	100%	0.0	99.40%	1452	MK811206.1
Uncultured Asaia sp. clone Lab 16S ribosomal RNA gene, partial sequence	uncultured Asaia sp.	2399	2399	100%	0.0	99.40%	1452	MK598732.1
Proteobacterium symbiont of Nilaparvata lugens clone B31 16S ribosomal RNA gene, partial sequence	proteobacterium symb...	2394	2394	100%	0.0	99.32%	1452	FJ774959.1
Uncultured bacterium clone Asaia sp. 16S ribosomal RNA gene, partial sequence	uncultured bacterium	2392	2392	100%	0.0	99.32%	1451	MK814862.1
Uncultured Asaia sp. clone donor-WBPH 16S ribosomal RNA gene, partial sequence	uncultured Asaia sp.	2388	2388	100%	0.0	99.24%	1453	MN094401.1
Uncultured bacterium clone Asaia sp. 16S ribosomal RNA gene, partial sequence	uncultured bacterium	2388	2388	100%	0.0	99.24%	1452	MK811207.1
Uncultured Asaia sp. clone recipient-WBPH 16S ribosomal RNA gene, partial sequence	uncultured Asaia sp.	2383	2383	100%	0.0	99.17%	1452	MN094403.1
Uncultured Acetobacteraceae bacterium clone NeHD1Act2 16S ribosomal RNA gene, partial sequence	uncultured Acetobacte...	2322	2322	100%	0.0	98.34%	1411	JQ726821.1
Uncultured bacterium partial 16S rRNA gene, clone 3_F01	uncultured bacterium	2322	2322	100%	0.0	98.34%	1368	FN421695.1
Uncultured Asaia sp. clone OTU108 16S ribosomal RNA gene, partial sequence	uncultured Asaia sp.	2316	2316	100%	0.0	98.26%	1411	OR045520.1
Uncultured Acetobacteraceae bacterium clone NeHD1Act3 16S ribosomal RNA gene, partial sequence	uncultured Acetobacte...	2316	2316	100%	0.0	98.26%	1411	JQ726822.1
Uncultured Acetobacteraceae bacterium clone NeHD1Act1 16S ribosomal RNA gene, partial sequence	uncultured Acetobacte...	2316	2316	100%	0.0	98.26%	1411	JQ726820.1
Asaia krungthepensis strain G3-3-08 16S ribosomal RNA gene, partial sequence	Asaia krungthepensis	2244	2244	100%	0.0	97.28%	1361	FJ816021.1
Asaia lannensis strain W4 16S ribosomal RNA gene, partial sequence	Asaia lannensis	2239	2239	100%	0.0	97.20%	1365	MF777040.1

(**B**) rRNA/ITS databases

Description	Scientific Name	Max Score	Total Score	Query Cover	E value	Per. Ident	Acc. Len	Accession
Asaia lannensis strain NBRC 102526 16S ribosomal RNA, partial sequence	Asaia lannensis	2239	2239	100%	0.0	97.20%	1414	NR_114144.1
Asaia lannensis strain AB92 16S ribosomal RNA, partial sequence	Asaia lannensis	2239	2239	100%	0.0	97.20%	1411	NR_041564.1
Asaia bogorensis NBRC 16594 16S ribosomal RNA, partial sequence	Asaia bogorensis ...	2228	2228	100%	0.0	97.05%	1414	NR_113849.1
Asaia prunellae JCM 25354 strain T-153 16S ribosomal RNA, partial sequence	Asaia prunellae J...	2228	2228	100%	0.0	97.05%	1417	NR_112880.1
Asaia bogorensis strain 71 16S ribosomal RNA, partial sequence	Asaia bogorensis	2228	2228	100%	0.0	97.05%	1411	NR_024728.1
Asaia krungthepensis strain NBRC 100057 16S ribosomal RNA, partial sequence	Asaia krungthepe...	2226	2226	100%	0.0	96.98%	1414	NR_113878.1
Asaia krungthepensis strain AA08 16S ribosomal RNA, partial sequence	Asaia krungthepe...	2224	2224	100%	0.0	96.98%	1411	NR_024810.1
Asaia spathodeae NBRC 105894 16S ribosomal RNA, partial sequence	Asaia spathodea...	2222	2222	100%	0.0	96.98%	1414	NR_114292.1
Asaia spathodeae NBRC 105894 strain GB23-2 16S ribosomal RNA, partial sequence	Asaia spathodea...	2222	2222	100%	0.0	96.98%	1414	NR_112953.1
Asaia platycodi JCM 25414 strain T-683 16S ribosomal RNA, partial sequence	Asaia platycodi J...	2222	2222	100%	0.0	96.98%	1434	NR_112879.1
Asaia siamensis strain NBRC 16457 16S ribosomal RNA, partial sequence	Asaia siamensis	2217	2217	100%	0.0	96.90%	1414	NR_113845.1
Asaia siamensis strain S60-1 16S ribosomal RNA, partial sequence	Asaia siamensis	2217	2217	100%	0.0	96.90%	1411	NR_024738.1
Asaia astilbis JCM 15831 strain T-6133 16S ribosomal RNA, partial sequence	Asaia astilbis JC...	2211	2211	100%	0.0	96.83%	1407	NR_122089.1
Swaminathania salitolerans strain PA 51 16S ribosomal RNA, partial sequence	Swaminathania s...	2180	2180	100%	0.0	96.39%	1455	NR_025217.1
Neoasaia chiangmaiensis strain NBRC 101099 16S ribosomal RNA, partial sequence	Neoasaia chiang...	2176	2176	99%	0.0	96.50%	1414	NR_113975.1
Neoasaia chiangmaiensis strain AC28 16S ribosomal RNA, partial sequence	Neoasaia chiang...	2170	2170	99%	0.0	96.43%	1411	NR_113007.1
Kozakia baliensis strain NBRC 16664 16S ribosomal RNA, partial sequence	Kozakia baliensis	2167	2167	100%	0.0	96.22%	1418	NR_113858.1
Kozakia baliensis strain Yo-3 16S ribosomal RNA, partial sequence	Kozakia baliensis	2167	2167	100%	0.0	96.22%	1415	NR_024773.1
Acetobacter lovaniensis strain NBRC 13753 16S ribosomal RNA, partial sequence	Acetobacter lova...	2154	2154	100%	0.0	96.07%	1415	NR_113632.1

Supplementary Figure 2. Phylogenetic tree based on 16S rRNA gene sequences from the six isolates (EV15G, EV15P, EV16GL, EV16GM, EV16P^T, EV17) and closely related strains. The tree was reconstructed using the neighbour-joining method in the MEGAX. Filled circles indicate branches that were also recovered using the maximum-likelihood method. Numbers at the branching points indicate bootstrap values (expressed as percentages of 1000 replications), and only those > 50 % are shown. *Acetobacter cerevisiae* LMG 1625^T (NR_118162) was used as an outgroup. Bar, 0.005 substitutions per nucleotide position.

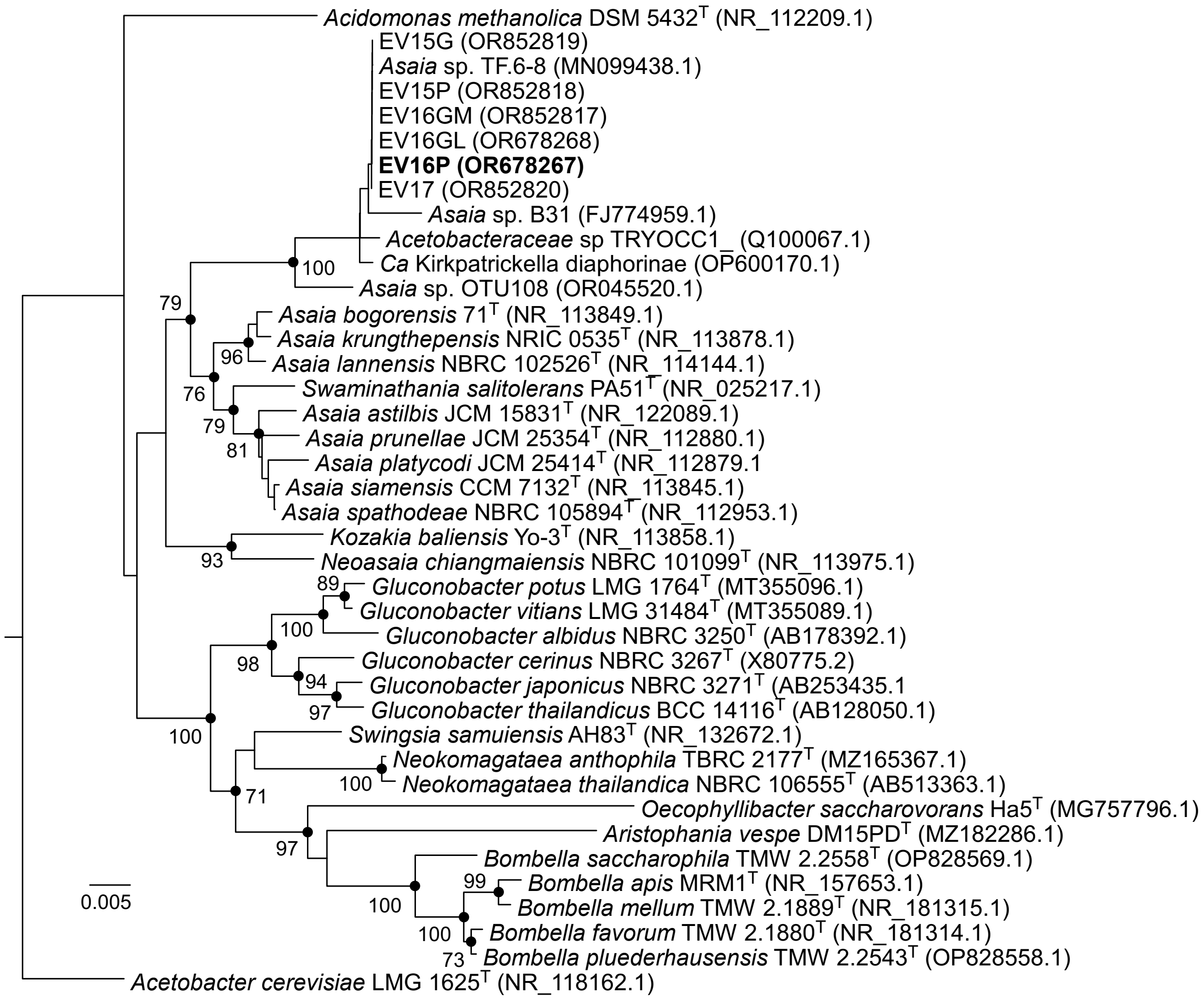

Supplementary Figure 3. Enterobacterial Repetitive Intergenic Consensus (ERIC) PCR profile of the six isolates. ERIC PCR was performed following the protocol described by De Bruijn [30].

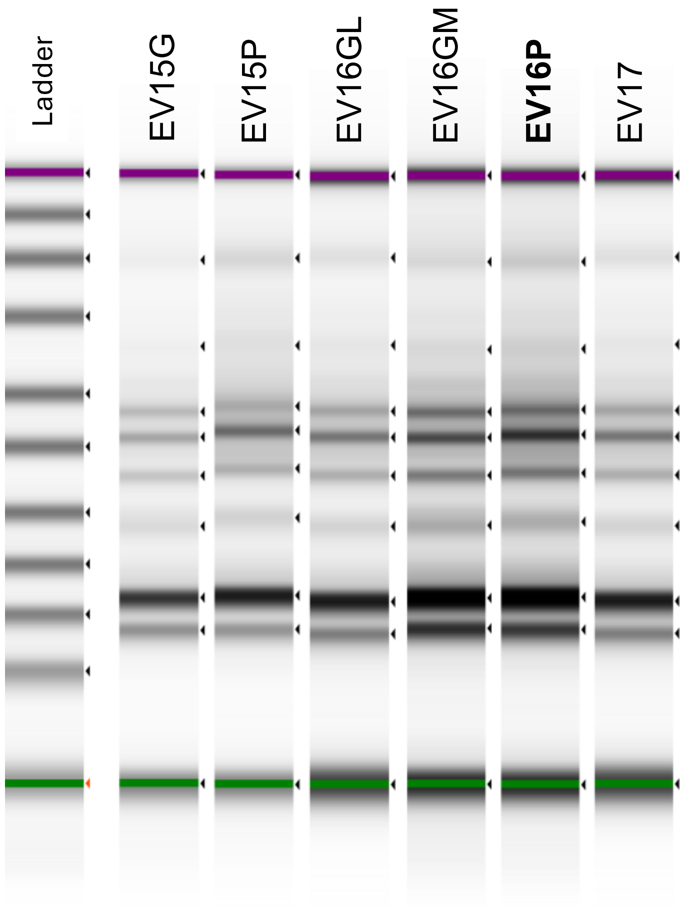

Supplementary Figure 4. Matrix of comparison for (**A**) average nucleotide identity based on BLAST (ANIb), (**B**) digital DNA–DNA hybridisation (dDDH), (**C**) average amino acid identity (AAI), and (**D**) percentage of conserved proteins (POCP). The pair comparisons include EV16P^T and the related closest type strains within the *Acetobacteraceae,* which are *Asaia astilbis* (n. 4, details in **Supplementary Table 1**), *A. bogorensis* (n. 5), *A. krungthepensis* (n. 6), *A. lannensis* (n. 7), *A. platycodi* (n. 8), *A. prunellae* (n. 9), *A. siamensis* (n. 10), *A. spathodeae* (n. 11), *Kozakia baliensis* (n. 23), *Swaminathania salitolerans* (n. 28). The symbiont of the hemipteran *Diaphorina citri*, *Ca*. Kirkpatrickella diaphorinae, was also included (n. 30).

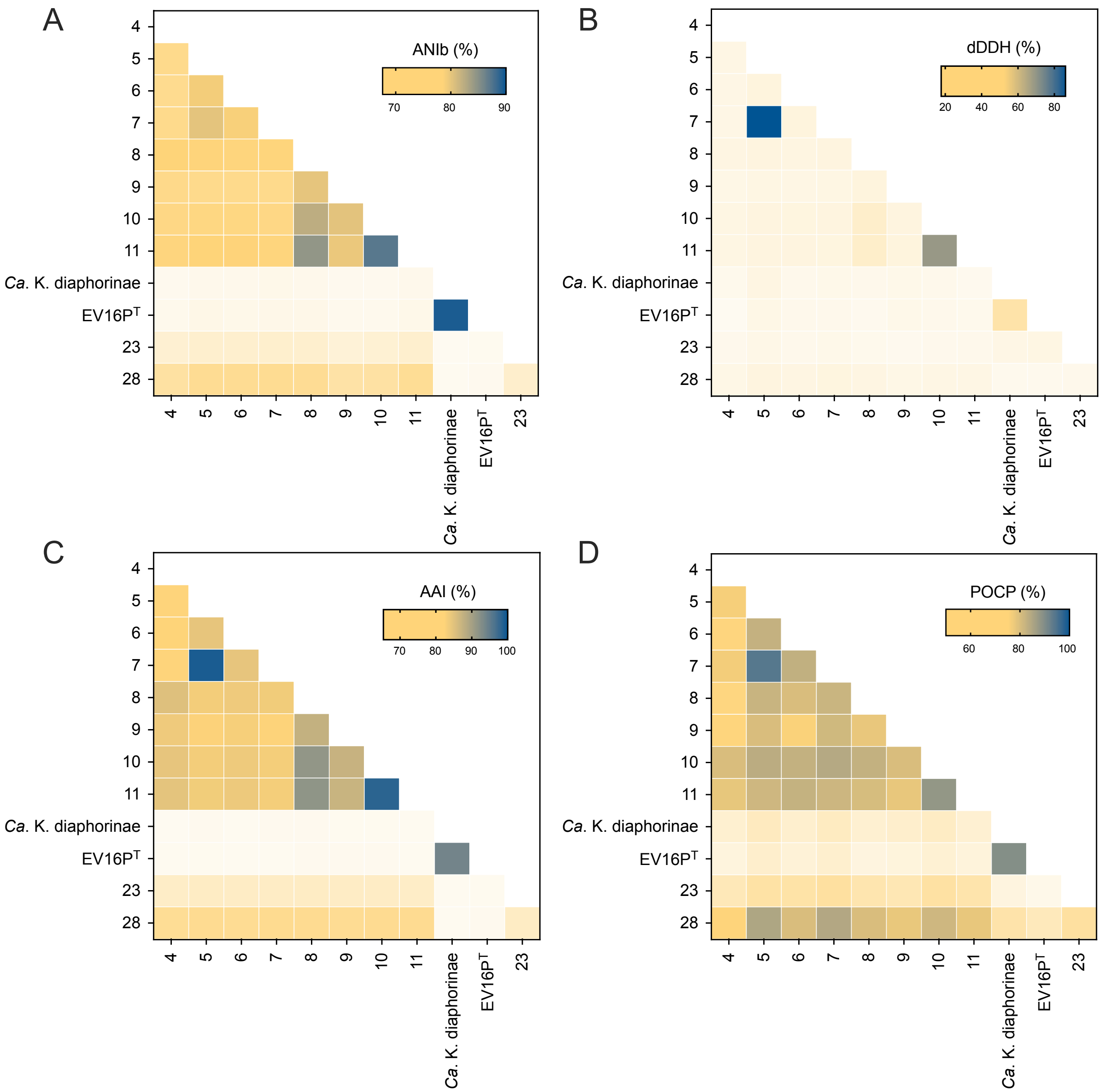

Supplementary Figure 5. Relationship between genome distance metrics for the type strains of close relatives calculated using the EV16P^T strain as the primary reference. (**A**) Plot of metrics based on nucleotide sequences, average nucleotide identity (gANI) and alignment fractions (AF). (**B**) Plot of metrics based on protein, percentage of conserved proteins (POCP) and average amino acid identity (AAI).

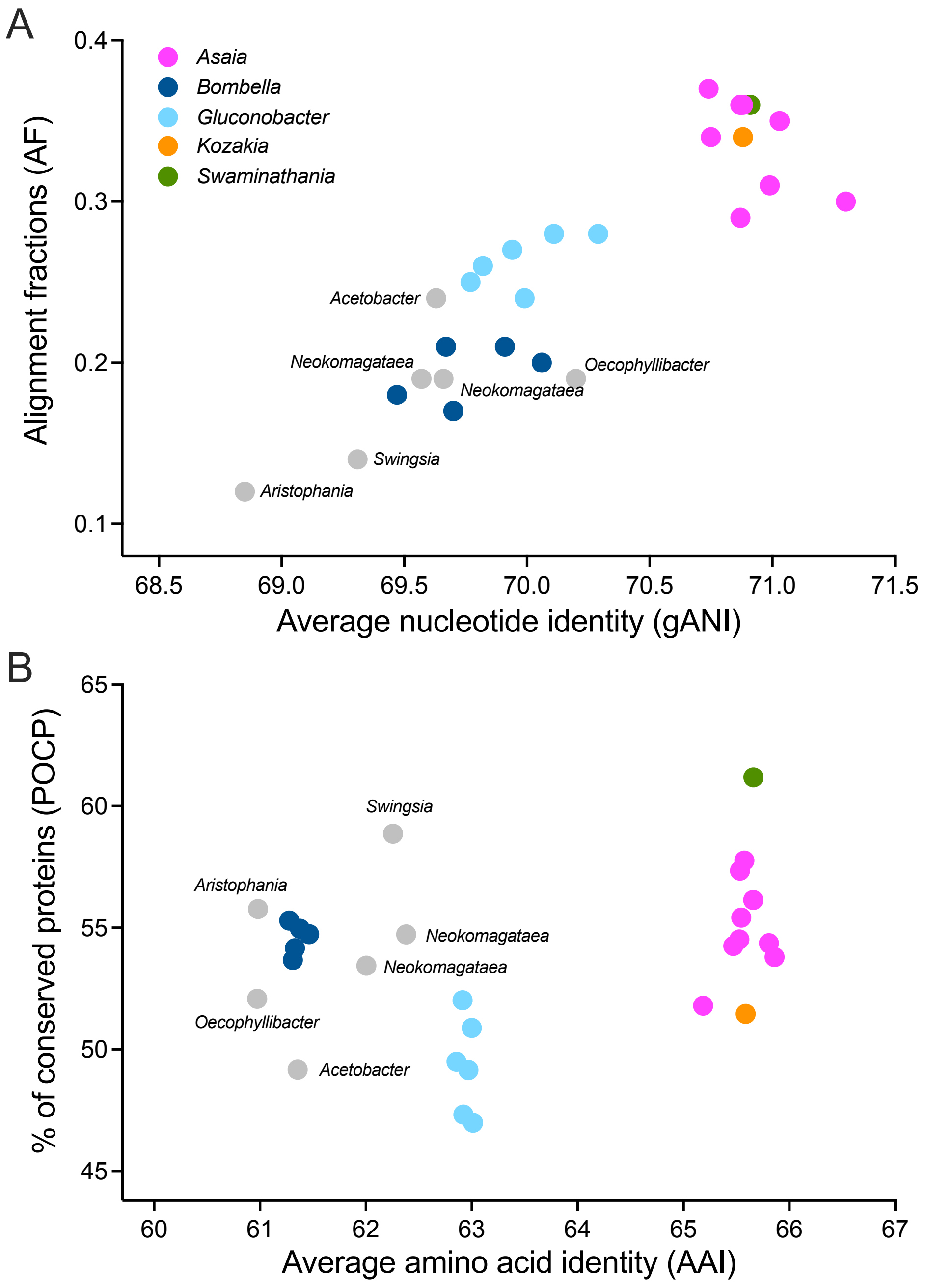

Supplementary Figure 6. GC-FID and -MS results of the cellular fatty acid for (**A**) EV16P^T, (**B**) *Kozakia baliensis* NRIC 0488^T (LMG 21812^T; [21]), (**C**) *Asaia bogorensis* 71^T (LMG 21650^T; [7]), and (**D**) *Swaminathania salitolerans* LMG 21291^T [27]. Analyses were performed by DSMZ.

(A)

RT [min]	Rfact	Area	ECL	ΔECL	Percent	Naming MIDI/TSBA6	GC-MS
2.707		0.4321					
3.320	1.1315	5.7319	10.913	-0.001		Summed feature 2 (12:0 aldehyde ? or unknown 10.928)	not confirmed
4.165	1.0712	0.6633	11.996	-0.004	0.1	12:0	OK
5.102	1.0316	11.3485	12.928	-0.008	2.5	13:1 at 12-13	Identified as an aldehyde
6.146	1.0009	0.5166	13.804				
6.379	1.0044	0.8522	13.997	-0.003	0.2	14:0	OK
7.068	0.9753	0.4043	14.499	-0.003	0.1	unknown 14.502	Identified as 14:0 DMA
7.680	0.9850	5.1234	14.945				
8.048	0.9701	0.7307	15.195	-0.008	0.2	14:0 2OH	OK
8.473	0.9681	9.0655	15.479	-0.001	2.0	Summed feature 2 (16:1 iso I/14:0 3OH)	Identified as 14:0 3OH
8.971	0.9658	1.6857	15.812	-0.010	0.4	Summed feature 3 (16:1 w7c/16:1 w6c)	Identified as 16:1 w7c
9.016	0.9656	0.9170	15.842	-0.010	0.2	Summed feature 3 (16:1 w6c/16:1 w7c)	Identified as 16:1 w6c
9.182	0.9648	0.4531	15.953				
9.250	0.9645	86.7616	15.999	-0.001	19.3	16:0	OK
9.913	0.9630	0.5095	16.422	0.006	0.1	Summed feature 9 (17:1 iso w9c or16:0 10-methyl)	Identified as 17:1 ISO w5c
10.053	0.9643	0.5678	16.512	0.009	0.1	15:0 3OH	Identified as 16:0 DMA
10.550	0.9689	4.6245	16.829	0.011	1.0	17:1 w7c	Identified as 17:1 w6c
10.814	0.9714	1.0435	16.998	-0.002	0.2	17:0	OK
11.175	0.9610	16.1382	17.224	-0.009	3.6	16:0 2OH	OK
11.512	0.9599	14.2990	17.434		3.2		Identified as 16: 3OH
11.631	0.9595	6.6416	17.508				
12.127	0.9578	117.6773	17.818	-0.005	26.1	Summed feature 8 (18:1 w7c or 18:1 w6c)	Identified as 18:1 w7c
12.190	0.9576	5.0862	17.858	0.000	1.1	Summed feature 8 (18:1 w6c or 18:1 w7c)	Identified as 18:1 w6c
12.411	0.9568	19.7419	17.996	-0.004	4.4	18:0	OK
12.534	0.9676	0.9456	18.073	-0.008	0.2	18:1 w7c 11-methyl	OK
12.938	0.9661	4.5741	18.326		1.0		Identified as a 18:1 DMA, π-bond unclear
13.847	0.9628	100.5944	18.895	-0.007	22.3	19:0 cyclo w8c	Identified as 19:0 cyclo w6c
13.965	0.9624	0.5986	18.969				
14.127	0.9737	4.9292	19.071				
14.201	0.9734	1.9977	19.117				
14.533	0.9721	1.8789	19.327				
14.865	0.9708	3.5317	19.537				
15.175	0.9696	0.8549	19.732	0.000		20:2 w6,9c	not confirmed
15.318	0.9691	0.7149	19.823	-0.008	0.2	20:1 w7c	Identified as 18:0 3OH
15.739		0.4012					
15.868		51.7278			11.5		Identified as 19:1 2OH, π-bond unclear
16.289		1.6790					
16.619		0.5358					
16.782		0.4057					
17.152		1.6642	3.579				
17.389		1.0266	4.731				
18.244		1.4946	3.892				
18.488		0.7569	2.144				
18.780		0.4446	1.815				
		**431.2033**					

(B)

RT [min]	Rfact	Area	ECL	ΔECL	Percent	Naming MIDI/TSBA6	GC-MS
2.266		0.2186					
2.489		0.1031					
2.710		0.3279					
3.321	1.1316	0.5428	10.915	0.001	1.7	Summed feature 2 (12:0 aldehyde ? or unknown 10.928)	Identified as 12:0 Aldehyde
5.101	1.0316	0.7079	12.927	-0.009		13:1 at 12-13	not confirmed
6.378	1.0044	0.4003	13.996	-0.004	1.3	14:0	OK
7.679	0.9850	0.4130	14.944				
8.051	0.9700	0.6609	15.197	-0.006	2.1	14:0 2OH	OK
8.468	0.9681	0.2609	15.476	-0.004	0.8	Summed feature 2 (16:1 iso I/14:0 3OH)	Identified as 14:0 3OH
8.968	0.9658	0.2184	15.810	-0.012	0.7	Summed feature 3 (16:1 w7c/16:1 w6c)	Identified as 16:1 w7c
9.247	0.9645	6.0071	15.997	-0.003	18.8	16:0	OK
10.815	0.9714	0.1899	16.999	-0.001	0.6	17:0	OK
11.178	0.9610	1.0082	17.225	-0.008	3.2	16:0 2OH	OK
12.120	0.9578	20.4604	17.814	-0.009	64.1	Summed feature 8 (18:1 w7c or 18:1 w6c)	Identified as 18:1 w7c
12.409	0.9568	1.0522	17.994	-0.006	3.3	18:0	OK
13.842	0.9628	1.1286	18.892	-0.010	3.5	19:0 cyclo w8c	Identified as 19:0 cyclo w6c
15.865		0.9319					
18.510		0.1323					
18.522		0.0897					
19.148		0.1078					
19.158		0.0633					
19.209		0.3173					
19.480		0.1221					
		**33.0506**					

(C)

RT [min]	Rfact	Area	ECL	ΔECL	Percent	Naming MIDI/TSBA6	GC-MS
2.115		0.2244					
2.264		0.1591					
2.707		0.6667					
2.763		0.1730					
3.054	1.1021	0.2723	10.467				
3.320	1.1315	2.0051	10.913	-0.001		Summed feature 2 (12:0 aldehyde ? or unknown 10.928)	not confirmed
3.704	1.0494	0.3593	11.417	-0.005	0.2	10:0 3OH	OK
4.165	1.0712	0.3181	11.996	-0.004	0.2	12:0	OK
5.100	1.0316	4.1457	12.926	-0.010		13:1 at 12-13	not confirmed
5.378	0.9893	0.1650	13.169	-0.008	0.1	12:0 2OH	OK
6.151	1.0010	0.1551	13.808				
6.305	1.0033	0.1711	13.935				
6.379	1.0044	1.1848	13.997	-0.003	0.7	14:0	OK
7.679	0.9850	2.0515	14.944				
7.753	0.9862	0.2345	14.998	-0.002	0.1	15:0	OK
8.048	0.9701	8.1934	15.195	-0.008	5.1	14:0 2OH	OK
8.472	0.9681	2.0455	15.479	-0.001	1.3	Summed feature 2 (16:1 iso I/14:0 3OH)	Identified as 14:0 3OH
8.967	0.9658	0.6333	15.809	-0.013	0.4	Summed feature 3 (16:1 w7c/16:1 w6c)	Identified as 16:1 w7c
9.174	0.9649	0.1686	15.948				
9.246	0.9645	20.6933	15.996	-0.004	12.9	16:0	OK
10.053	0.9643	0.1911	16.512	0.009		15:0 3OH	not confirmed
10.589	0.9693	0.3784	16.854	-0.006	0.2	17:1 w6c	OK
10.813	0.9714	0.4507	16.997	-0.003	0.3	17:0	OK
11.174	0.9610	10.7987	17.223				
11.631	0.9595	1.7830	17.508				
12.126	0.9578	109.4068	17.818	-0.005	68.1	Summed feature 8 (18:1 w7c or 18:1 w6c)	Identified as18:1 w7c
12.281	0.9573	0.1538	17.914	-0.005	0.1	18:1 w5c	OK
12.409	0.9568	3.5017	17.994	-0.006	2.2	18:0	OK
13.841	0.9628	1.3876	18.891	-0.011	0.9	19:0 cyclo w8c	Identified as 19:0 cyclo w6c
14.127	0.9737	1.8711	19.071				
14.528	0.9721	0.7166	19.324				
14.863	0.9708	0.5686	19.535		0.4		Identified as 18:0 3OH
15.318	0.9691	0.1557	19.823	-0.008		20:1 w7c	not confirmed
15.865		10.9962			6.8		Identified as 19:1 2OH, π-bond position unclear
16.347		0.2656					
17.153		0.4989					
18.238		0.5855					
18.497		0.2881					
18.785		0.6739					
18.995		1.5222					
		**174.1604**					

(D)

RT [min]	Rfact	Area	ECL	ΔECL	Percent	Naming MIDI/TSBA6	GC-MS
2.484		0.1132					
2.707		0.3345					
3.320	1.1315	1.5398	10.913	-0.001		Summed feature 2 (12:0 aldehyde ? or unknown 10.928)	not confirmed
4.165	1.0712	0.1702	11.996	-0.004	0.1	12:0	OK
5.099	1.0315	3.3585	12.925	-0.011		13:1 at 12-13	not confirmed
5.383	0.9894	0.1357	13.173	-0.004	0.1	12:0 2OH	OK
6.150	1.0010	0.2068	13.807				
6.378	1.0044	1.3674	13.996	-0.004	1.2	14:0	OK
7.680	0.9850	1.5805	14.945				
7.756	0.9714	0.1678	15.000	0.000	0.1	15:0	OK
8.048	0.9701	6.5366	15.195	-0.008	5.6	14:0 2OH	OK
8.472	0.9681	1.2536	15.479	-0.001	1.1	Summed feature 2 (16:1 iso I/14:0 3OH)	Identified as 14:0 3OH
8.967	0.9658	0.5809	15.809	-0.013	0.5	Summed feature 3 (16:1 w7c/16:1 w6c)	Identified as 16:1 w7c
9.177	0.9648	0.1797	15.950				
9.246	0.9645	16.4656	15.996	-0.004	14.1	16:0	OK
10.057	0.9643	0.1623	16.514				
10.590	0.9693	0.3390	16.855	-0.005		17:1 w6c	not confirmed
10.808	0.9713	0.2919	16.994	-0.006	0.3	17:0	OK
11.174	0.9610	6.2353	17.223		5.3		Identified as 16:0 2OH
11.631	0.9595	1.0459	17.508		0.9		Identified as 16:0 3OH
12.123	0.9578	64.7418	17.816	-0.007	55.5	Summed feature 8 (18:1 w7c or 18:1 w6c)	Identified as 18:1 w7c
12.409	0.9568	1.9930	17.994	-0.006	1.7	18:0	OK
12.526	0.9676	0.1920	18.068	-0.013		18:1 w7c 11-methyl	not confirmed
13.841	0.9628	6.6548	18.891	-0.011	5.7	19:0 cyclo w8c	Identified as 19:0 cyclo w6c
14.128	0.9737	0.5398	19.071				
14.526	0.9721	0.6524	19.323				
14.866	0.9708	0.4114	19.537				
15.173	0.9696	0.2304	19.731	-0.001		20:2 w6,9c	not confirmed
15.864		9.0016			7.7		Identified as 19:1 2OH, π-bond position unclear
16.280		0.5396					
17.152		0.5850					
18.249		0.5431					
18.490		0.2858					
18.780		0.2087					
19.146		0.4662					
		**117.0331**					

Supplementary Figure 7. Results of polar lipids analysis conducted by DSMZ on (**A**) EV16P^{T}, (**B**) *Kozakia baliensis* NRIC 0488^{T} (LMG 21812^{T}; [21]), (**C**) *Asaia bogorensis* 71^{T} (LMG 21650^{T}; [7]), and (**D**) *Swaminathania salitolerans* LMG 21291^{T} [27].

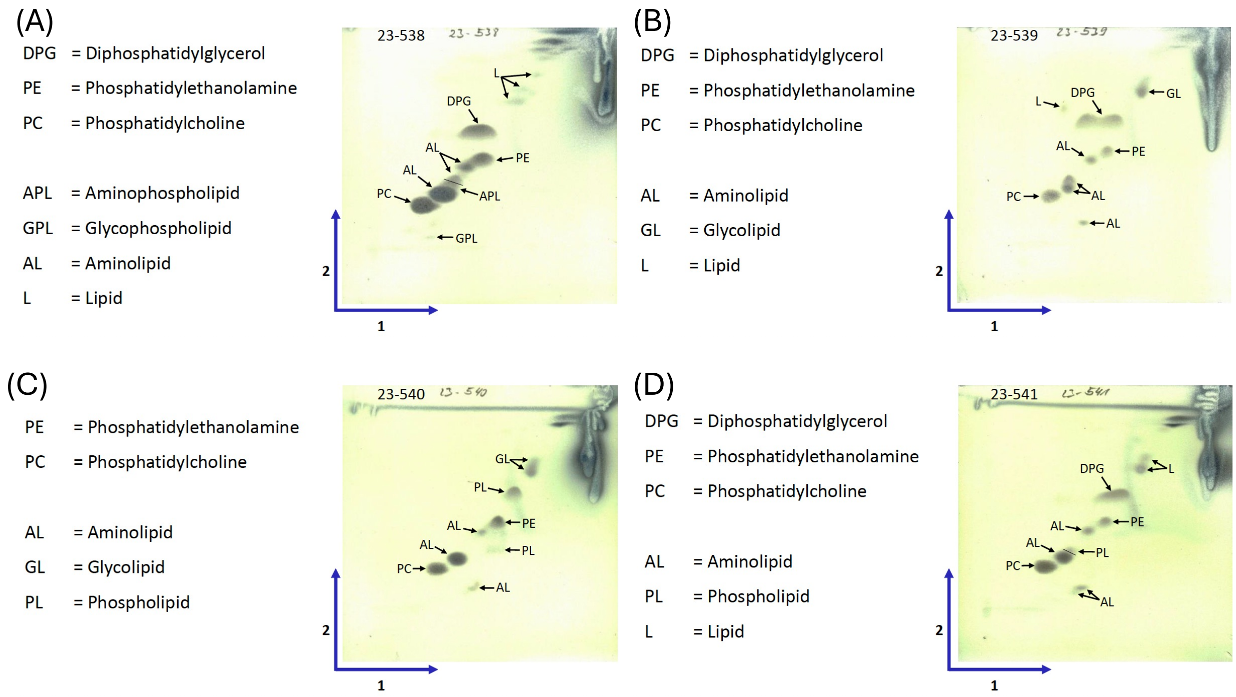

Supplementary Figure 8. Results of API ZYM kit for EV16P^{T}, *Asaia bogorensis* 71^{T} (LMG 21650^{T}; [7]), *Swaminathania salitolerans* LMG 21291^{T} [27], *Kozakia baliensis* NRIC 0488^{T} (LMG 21812^{T}; [21]). Colour changes have been interpreted as indicated by the manufacturer's instructions for a positive or negative activity of enzymes.

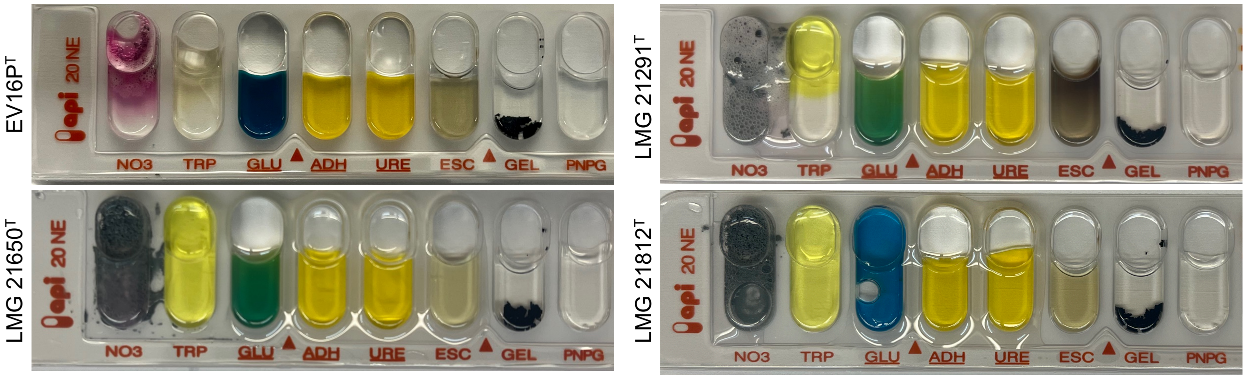

Supplementary Figure 9. Results of API ZYM kit for EV16P^{T}, *Asaia bogorensis* 71^{T} (LMG 21650^{T}; [7]), *Swaminathania salitolerans* LMG 21291^{T} [27], *Kozakia baliensis* NRIC 0488^{T} (LMG 21812^{T}; [21]). Colour changes have been interpreted as indicated by the manufacturer's instructions for a positive or negative activity of enzymes. 1: Control, 2: Alkaline phosphatase, 3: Esterase (C 4), 4: Esterase Lipase (C 8), 5: Lipase (C 14), 6: Leucine arylamidase, 7: Valine arylamidase, 8: Cystine arylamidase, 9: Trypsin, 10: α-chymotrypsin, 11: Acid phosphatase, 12: Naphthol-AS-BI-phosphohydrolase, 13: α-galactosidase, 14: ß-galactosidase, 15: ß-glucuronidase, 16: α-glucosidase, 17: ß-glucosidase, 18: N-acetyl-ß-glucosaminidase, 19: α-mannosidase, 20: α-fucosidase.

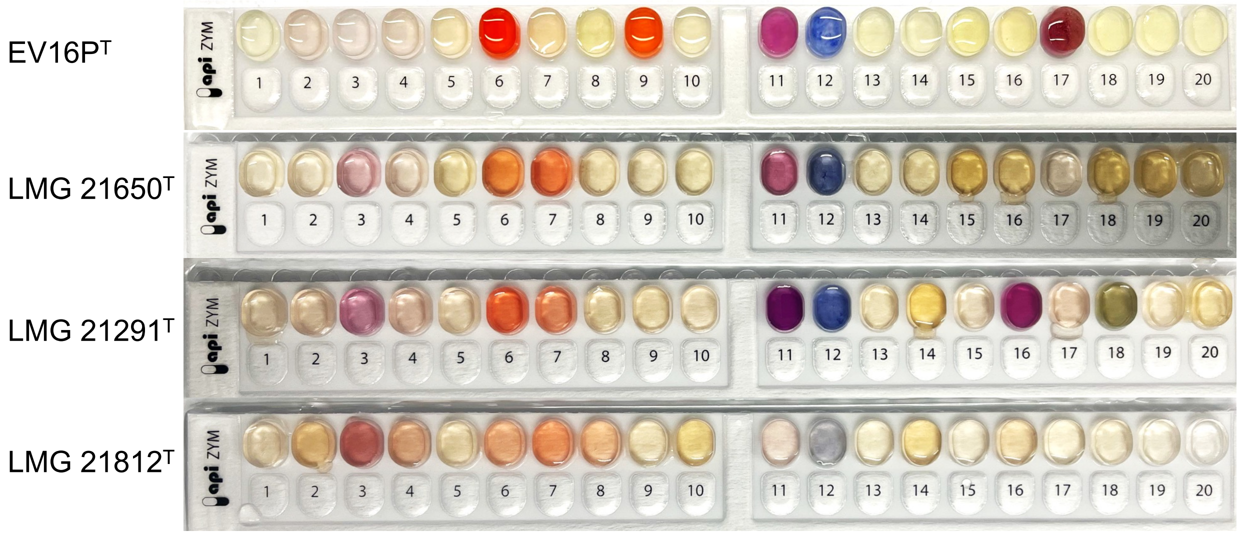

Supplementary Figure 10. Acid production from carbohydrates (wells 1-49) was assessed with API 50CH system for EV16P^T, *Asaia bogorensis* 71^T (LMG 21650^T; [7]), *Swaminathania salitolerans* LMG 21291^T [27], *Kozakia baliensis* NRIC 0488^T (LMG 21812^T; [21]). After 72 h of incubation, the colour changes (yellow, orange, or brown) with respect to control (red, well 0) were registered.

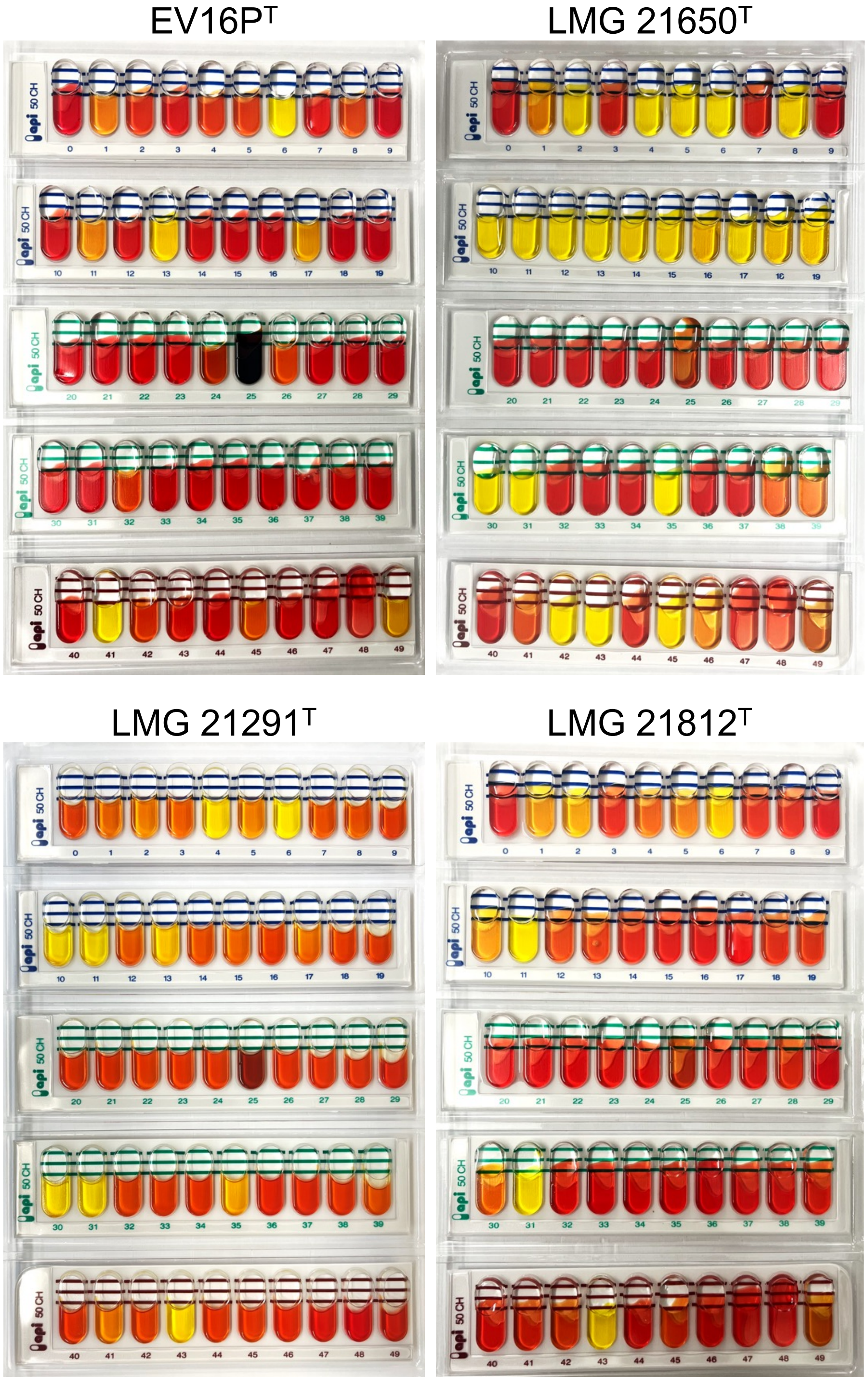

References

1. **Gonella E, Crotti E, Mandrioli M, Daffonchio D, Alma A**. *Asaia* symbionts interfere with infection by Flavescence dorée phytoplasma in leafhoppers. *J Pest Sci* 2018;91:1033–1046.
2. **Crotti E, Damiani C, Pajoro M, Gonella E, Rizzi A, *et al.*** *Asaia*, a versatile acetic acid bacterial symbiont, capable of cross-colonizing insects of phylogenetically distant genera and orders. *Environ Microbiol* 2009;11:3252–3264.
3. **Cleenwerck I, Vandemeulebroecke K, Janssens D, Swings J**. Re-examination of the genus *Acetobacter*, with descriptions of *Acetobacter cerevisiae* sp. nov. and *Acetobacter malorum* sp. nov. *Int J Syst Evol Microbiol* 2002;52:1551–1558.
4. **Yamashita S, Uchimura T, Komagata K**. Emendation of the genus *Acidomonas* Urakami, Tamaoka, Suzuki and Komagata 1989. *Int J Syst Evol Microbiol* 2004;54:865–870.
5. **Guzman J, Won M, Poehlein A, Sombolestani AS, Mayorga-Ch D, *et al.*** *Aristophania vespae* gen. nov., sp. nov., isolated from wasps, is related to *Bombella* and *Oecophyllibacter*, isolated from bees and ants. *Int J Syst Evol Microbiol* 2023;73:005699.
6. **Suzuki R, Zhang Y, Iino T, Kosako Y, Komagata K, *et al.*** *Asaia astilbes* sp. nov., *Asaia platycodi* sp. nov., and *Asaia prunellae* sp. nov., novel acetic acid bacteria isolated from flowers in Japan. *J Gen Appl Microbiol* 2010;56:339–346.
7. **Yamada Y, Katsura K, Kawasaki H, Widyastuti Y, Saono S, *et al.*** *Asaia bogorensis* gen. nov., sp. nov., an unusual acetic acid bacterium in the alpha-Proteobacteria. *Int J Syst Evol Microbiol* 2000;50:823–829.
8. **Kawai M, Higashiura N, Hayasaki K, Okamoto N, Takami A, *et al.*** Complete genome and gene expression analyses of *Asaia bogorensis* reveal unique responses to culture with mammalian cells as a potential opportunistic human pathogen. *DNA Research* 2015;22:357–366.
9. **Yukphan P, Potacharoen W, Tanasupawat S, Tanticharoen M, Yamada Y**. *Asaia krungthepensis* sp. nov., an acetic acid bacterium in the α-Proteobacteria. *Int J Syst Evol Microbiol* 2004;54:313–316.
10. **Malimas T, Yukhpan P, TakahashiI M, Kaneyasu M, Potacharoen W, *et al.*** *Asaia lannaensis* sp. nov., a new acetic acid bacterium in the *Alphaproteobacteria*. *Biosci Biotechnol Biochem* 2008;72:666–671.
11. **Katsura K, Kawasaki H, Potacharoen W, Saono S, Seki T, *et al.*** *Asaia siamensis* sp. nov., an acetic acid bacterium in the alpha-proteobacteria. *Int J Syst Evol Microbiol* 2001;51:559–563.
12. **Kommanee J, Tanasupawat S, Yukphan P, Malimas T, Muramatsu Y, *et al.*** *Asaia spathodeae* sp. nov., an acetic acid bacterium in the α-Proteobacteria. *J Gen Appl Microbiol* 2010;56:81–87.
13. **Yun J-H, Lee J-Y, Hyun D-W, Jung M-J, Bae J-W**. *Bombella apis* sp. nov., an acetic acid bacterium isolated from the midgut of a honey bee. *Int J Syst Evol Microbiol* 2017;67:2184–2188.
14. **Hilgarth M, Redwitz J, Ehrmann MA, Vogel RF, Jakob F**. *Bombella favorum* sp. nov. and *Bombella mellum* sp. nov., two novel species isolated from the honeycombs of *Apis mellifera*. *Int J Syst Evol Microbiol* 2021;71:004633.

15. **Härer L, Stýblová S, Ehrmann MA**. *Bombella pluederhausensis* sp. nov., *Bombella pollinis* sp. nov., *Bombella saccharophila* sp. nov. and *Bombella dulcis* sp. nov., four *Bombella* species isolated from the environment of the western honey bee *Apis mellifera*. *Int J Syst Evol Microbiol* 2023;73:005927.
16. **Yukphan P, Takahashi M, Potacharoen W, Tanasupawat S, Nakagawa Y, *et al.*** *Gluconobacter albidus* (ex Kondo and Ameyama 1958) sp. nov., nom. rev., an acetic acid bacterium in the alpha-Proteobacteria. *J Gen Appl Microbiol* 2004;50:235–242.
17. **Katsura K, Yamada Y, Uchimura T, Komagata K**. *Gluconobacter asaii* Mason and Claus 1989 is a junior subjective synonym of *Gluconobacter cerinus* Yamada and Akita 1984. *Int J Syst Evol Microbiol* 2002;52:1635–1640.
18. **Malimas T, Yukphan P, Takahashi M, Muramatsu Y, Kaneyasu M, *et al.*** *Gluconobacter japonicus* sp. nov., an acetic acid bacterium in the *Alphaproteobacteria*. *Int J Syst Evol Microbiol* 2009;59:466–471.
19. **Sombolestani AS, Cleenwerck I, Cnockaert M, Borremans W, Wieme AD, *et al.*** Characterization of novel *Gluconobacter* species from fruits and fermented food products: *Gluconobacter cadivus* sp. nov., *Gluconobacter vitians* sp. nov. and *Gluconobacter potus* sp. nov. *Int J Syst Evol Microbiol* 2019;71:004751.
20. **Tanasupawat S, Thawai C, Yukphan P, Moonmangmee D, Itoh T, *et al.*** *Gluconobacter thailandicus* sp. nov., an acetic acid bacterium in the alpha-Proteobacteria. *J Gen Appl Microbiol* 2004;50:159–167.
21. **Lisdiyanti P, Kawasaki H, Widyastuti Y, Saono S, Seki T, *et al.*** *Kozakia baliensis* gen. nov., sp. nov., a novel acetic acid bacterium in the alpha-Proteobacteria. *Int J Syst Evol Microbiol* 2002;52:813–818.
22. **Schmid J, Koenig S, Pick A, Steffler F, Yoshida S, *et al.*** Draft genome sequence of *Kozakia baliensis* SR-745, the first sequenced *Kozakia* strain from the family *Acetobacteraceae*. *Genome Announc* 2014;2:10.1128/genomeA.00594-14.
23. **Yukphan P, Malimas T, Potacharoen W, Tanasupawat S, Tanticharoen M, *et al.*** *Neoasaia chiangmaiensis* gen. nov., sp. nov., a novel osmotolerant acetic acid bacterium in the alpha-Proteobacteria. *J Gen Appl Microbiol* 2005;51:301–311.
24. **Charoenyingcharoen P, Yukphan P, Malimas S, Likhitrattanapisal S, Tanasupawat S, *et al.*** *Neokomagataea anthophila* sp. nov., an osmotolerant acetic acid bacterium isolated in Thailand and emended description of the genus *Neokomagataea*. *Int J Syst Evol Microbiol* 2022;72:005428.
25. **Yukphan P, Malimas T, Muramatsu Y, Potacharoen W, Tanasupawat S, *et al.*** *Neokomagataea* gen. nov., with descriptions of *Neokomagataea thailandica* sp. nov. and *Neokomagataea tanensis* sp. nov., osmotolerant acetic acid bacteria of the α-Proteobacteria. *Biosci Biotechnol Biochem* 2011;75:419–426.
26. **Chua K-O, See-Too W-S, Tan J-Y, Song S-L, Yong H-S, *et al.*** *Oecophyllibacter saccharovorans* gen. nov. sp. nov., a bacterial symbiont of the weaver ant *Oecophylla smaragdina*. *J Microbiology* 2020;58:988–997.
27. **Loganathan P, Nair S**. *Swaminathania salitolerans* gen. nov., sp. nov., a salt-tolerant, nitrogen-fixing and phosphate-solubilizing bacterium from wild rice (*Porteresia coarctata* Tateoka). *Int J Syst Evol Microbiol* 2004;54:1185–1190.
28. **Malimas T, Chaipitakchonlatarn W, Thi Lan Vu H, Yukphan P, Muramatsu Y, *et al.*** *Swingsia samuiensis* gen. nov., sp. nov., an osmotolerant acetic acid bacterium in the α-Proteobacteria. *J Gen Appl Microbiol* 2013;59:375–384.

29. **Henry E, Carlson CR, Kuo YW**. *Candidatus* Kirkpatrickella diaphorinae gen. nov., sp. nov., an uncultured endosymbiont identified in a population of *Diaphorina citri* from Hawaii. *Int J Syst Evol Microbiol* 2023;73:1–10.
30. **De Bruijn FJ**. Use of repetitive (repetitive extragenic palindromic and enterobacterial repetitive intergeneric consensus) sequences and the polymerase chain reaction to fingerprint the genomes of *Rhizobium meliloti* isolates and other soil bacteria. *Appl Environ Microbiol* 1992;58:2180–2187.
